# Engineered DNase-inactive Cpf1 variants to improve targeting scope for base editing in *E. coli*

**DOI:** 10.1016/j.synbio.2021.09.002

**Published:** 2021-09-24

**Authors:** Zehua Chen, Jinyuan Sun, Ying Guan, Ming Li, Chunbo Lou, Bian Wu

**Affiliations:** a CAS Key Laboratory of Microbial Physiological & Metabolic Engineering and State Key Laboratory of Microbial Resources, Institute of Microbiology, Chinese Academy of Sciences, Beijing, 100101, China; b College of Life Sciences, University of Chinese Academy of Sciences, Beijing, 100149, China; c Tsinghua University, Beijing, 100084, China

**Keywords:** Base editing, Directed evolution, Broad-spectrum dFnCpf1 (bsdFnCpf1), Multiple gene editing

## Abstract

The development of base editing (BE) technology has opened a new avenue for research studies in bacteriology, particularly for bacterial species in which the DNA double-strand breaks (DSBs) introduced by CRISPR/Cas system would lead to cell death. However, a major limitation of BE-mediated gene editing is the restricted editable sites in the target bacterial genome due to highly diverse genomic compositions, such as GC content. Herein, we developed a broad-spectrum DNase-inactive Cpf1 (dCpf1) variant from *Francisella novicida* (bsdFnCpf1) through directed evolution. The resulting optimized mutant showed a substantially expanded targeting range, including previously non-canonical protospacer-adjacent motifs (PAMs), especially the GC-rich PAMs. Cytidine deaminase APOBEC1 and uracil DNA glycosylase inhibitor (UGI) were fused with bsdFnCpf1 to achieve specific C to T mutations at multiple target sites with canonical or non-canonical PAMs in the *E. coli* genome without compromising cell growth. We anticipate that bsdFnCpf1 could be applied for multiplex gene regulation and BE in species that have been reported to be suitable for Cpf1.

## 1 Introduction

Gene editing has emerged as an important tool in bacteriological research studies, such as studies related to the genetics of bacterial pathogenicity and metabolic engineering for the production of biofuels or medicinal drugs [[1], [2], [3]]. As compared to traditional bacterial gene-editing methods, such as lambda-red assisted recombination and site-specific recombination [[4], [5], [6]], the CRISPR-Cas system is a highly efficient and convenient tool for marker-free and scar-free gene-editing [[7], [8], [9]]. However, in most bacterial species, DSBs generated by Cas nucleases would result in cell death or abnormal cell growth due to the absence of the NHEJ repair pathway [10,11]. Thus, the BE system, involving CRISPR-Cas proteins and cytosine/adenine deaminases, has been employed to obtain accurate and irreversible base substitutions (C-to-T or A-to-G) [12,13]. Apart from being free from the lethal effects of DSBs, this BE system is accurate and has demonstrated a high editing efficiency in numerous bacterial species, including *Staphylococcus aureus* [14]*, Pseudomonas* [15]*, Corynebacterium glutamicum* [16]*, Brucella melitensis* [17]*,* and *Escherichia coli* [17,18]. However, the limited availability of editable sites in the target bacterial genome and off-target activity have restrained the applicability of CRISPR-assisted multiplex BE system.

Cpf1 is an alternative to the commonly used CRISPR nuclease, Cas9. Due to its RNA endonuclease activity, Cpf1 has a certain advantage in multiplex gene targeting in the same cell [19,20]. Simultaneous manipulation of multiple genes is in high demand in system research studies, as it enables the investigation of overly complex interactions in genome-scale networks. In addition, the Cpf1 system displays enticing features, such as a concise crRNA (∼40 nt), low molecular weight, and low off-target activity [21,22]. These properties provide distinct advantages to multiplex gene editing and perturbation. Furthermore, the CRISPR/dCpf1 system could be repurposed for targeting genomic DNA without introducing DSBs. Thus, we envision that dCpf1 is an efficient tool with a high potential for multi-gene regulation and base editing in microorganisms.

Nevertheless, Cpf1 mediated gene editing essentially requires the recognition of a T-rich PAM of form 5′ -TTTV/TTV (V represents A, C, or G), which hinders its application in gene editing of GC-rich organisms [20]. To address this limitation, two *Acidaminococcus* sp. Cpf1 (AsCpf1) variants RVR and RR were initially engineered to recognize alternative PAMs, i.e., TATV and TYCV, respectively [23]. Later, the target range of AsCpf1 was further expanded to TTYN/VTTV/TRTV PAMs [24]. Besides, another widely used Cpf1 from *Francisella novicida* (FnCpf1), was engineered to identify non-canonical PAMs [25], however the -4 T preference in the PAM sequence was ignored in the study [26,27]. These studies have identified compensatory mutations that result in altered PAM specificity. The resulting Cpf1 variants maintained a thymine preference in at least one position of the PAM sequence. Although these studies have improved the applicability of Cpf1, multiple PAMs, especially most GC-rich PAM sequences, remain inaccessible. Thus, additional mutants with expanded targeting are still desired for applications demanding high targeting density and flexibility.

In this study, we designed a negative screening assay based on fluorescence for transcriptional repression by the CRISPR-dCpf1 system in *E. coli*. This screening assay was used to quantify the functional effects of dCpf1 mutants systematically. Then, directed evolution was performed to extend the PAM preference for dFnCpf1 to the GC-rich PAMs. The resulting subset of dFnCpf1 mutants exhibited higher recognition abilities and binding affinities for sites with non-canonical PAMs and retained robust activities on canonical TTTV PAMs. Through detailed characterization, a broad-spectrum dFnCpf1 mutant (referred to as bsdFnCpf1) was identified with a substantially expanded targeting range. Furthermore, we demonstrated that bsdFnCpf1 could be designed as cytosine base editor bsdFnCpf1-BE in multiplex genome editing in *E. coli* with higher efficiency and broader targeting range than wild-type (WT) dFnCpf1-BE and previously reported dCpf1-BE. As bsdFnCpf1 does not rely on any additional or host-dependent factors, it lends higher flexibility to the CRISPR-dCpf1 system in synthetic biological applications [[28], [29], [30]].

## 2 Materials and methods

### 2.1 Bacteria and culture conditions

The *E. coli* strain *DH5a* strain was used in this study. *E. coli* strain was cultured in LB (10 g/L tryptone, 5 g/L yeast extract, 10 g/L NaCl) or M9 media (12.8 g/L Na_2_HPO_4_·7H_2_O, 3 g/L KH_2_PO_4_, 0.5 g/L NaCl, 1.67 g/L NH_4_Cl, 1 mM thiamine hydrochloride, 0.4% glucose, 0.2% casamino acids, 2 mM MgSO_4_, 0.1 mM CaCl_2_). LB was used as the growth media. Cells for flow cytometric fluorescence analysis were cultured in M9 media. Ampicillin (100 g/mL, Inalco, Spain), chloramphenicol (34 g/mL, Inalco), and kanamycin (50 g/mL, Inalco) were used in this study. The bacterial culture was incubated at 37 °C on a microplate shaker set to 1000 rpm (All-Sheng, Hangzhou, China) or on a rotary shaker set to 200 rpm in a 10 mL tube containing 5 mL medium (Honor, Tianjin, China).

### 2.2 Plasmid construction

Gibson Assembly [31] or Golden Gate Assembly [32] was employed to construct plasmids used in this study. The plasmid sequences were confirmed via Sanger sequencing. The *dfncpf1* gene was mutated and inserted into the Repressor Generator Plasmid (RGP) containing a pTac inducible promoter, a p15A replication origin, and an ampicillin-selectable marker, which was constructed in our previous study [33]. The vector was used to control the inducible expression of dCpf1 enzymes. The crRNA plasmid contained a synthetic constitutive promoter J23119, a ColE1 replication origin, and a chloramphenicol-selectable marker for crRNA expression. The reporter plasmid contained a pSC101 replication origin, a kanamycin-selectable marker, and an *yfp* as the reporter gene regulated by a J23100 promoter. In the BE experiment, the *dfncpf1* gene was replaced by the base editor gene: *apobec1-dfncpf1-ugi*, *apobec1-bsdfncpf1-ugi,* and *apobec1-denascpf1-ugi*. The *ugi* and *apobec1* genes were synthesized by Genscript Inc. The sequences of all bacterial expression plasmids were provided in Supplementary Table 1.

### 2.3 Fluorescence measurement by flow cytometry

Bacterial cells were cultured overnight, diluted 196 times using the M9 medium containing three antibiotics, and later incubated for 3 h. After incubation, cells were diluted 1000 times in the M9 medium containing three antibiotics and 200 μM IPTG followed by shaking at 37 °C for 8 h. The optimal concentration of the inducer IPTG was determined by the repression curve of WT dFnCpf1, as described in previous work [26]. To stop protein expression prior to flow cytometry analysis, bacterial cells were diluted using PBS containing 2 mg/mL kanamycin. The fluorescence intensity of YFP was measured using a Calibur flow cytometer (BD Biosciences, CA, USA) with appropriate settings (FSC 440, SSC 260, FITC 480). Minimum 50,000 events were collected for each sample. The geometric mean of fluorescence intensity of each sample was analyzed using FlowJo software version 7.6.2 (Treestar, USA), and the autofluorescence of *E. coli* was subtracted for each sample.

### 2.4 High throughput screening for dCpf1 mutants

The *dFncpf1* gene was amplified by error-prone PCR using forward primer: EPPCR_FW, 5′-TCAAAACAAAGACAATTTGGCACAGATATCTATCAAATATCAAAATCA-3′ and reverse primer: EPPCR_RV, 5′-CCCTGATTAACTACGCTATCAATATAGC-3′. The mutation rate per round was approximately 0.15% in our preliminary experiment. Later, the PCR products were inserted into the modified RGP plasmid using the Golden Gate method. The resulting *dfncpf1* mutant libraries were transformed into the *E. coli DH5a* cells harboring the reporter plasmid and the crRNA plasmid. The transformants were cultured overnight (∼14 h), diluted, and induced by 200 μM IPTG for 6 h. Later, cells with relatively lower fluorescence (lower than an artificially defined threshold) were sorted into fresh LB medium using a BD Influx cell sorter (BD, USA). After 3 h of cell resuscitation, the sorted cells were plated on LB agar containing three antibiotics. The clones were picked and cultured further for flow cytometry (BD Fortessa, USA) based validation studies. Cells with relatively low fluorescence were sequenced and collected for the next round of mutant screening. The positive control (*E. coli DH5a* strain containing the pSC101-J23100-*yfp* plasmid) and the negative control (*E. coli DH5a* strain containing the pSC101-J23100*,* pColE1-J23119-crRNA*,* and p15A-pTac*-dfncpf1* plasmids) were used to set the appropriate gain for the fluorescence channel. Sequences of crRNAs and target sites were provided in Supplementary Table 2.

### 2.5 PAM preference profiles analysis

A randomized PAM library (NNNC) was constructed through PCR and Gibson ligation. The reporter plasmid was used as the template (forward primer: Random_PAM_FW, 5′-TGTCAACGGTCATAANNNCCGTGCGTGGCGAGGGTGAAGGCGCAACTAAT-3′ and reverse primer: Random_PAM_RV, 5′-TTATGACCGTTGACATCACCATCCAGT-3′). The 64 PAM plasmids were transformed separately into competent *E. coli DH5α* cells harboring dFnCpf1 mutants and crRNA plasmids. The fluorescence intensity of YFP was measured using a Calibur flow cytometer (BD Biosciences, CA, USA), and the data was analyzed using FlowJo software version 7.6.2 (Treestar, USA). The PAM preference profiles were analyzed and displayed using Matlab.

### 2.6 Deep sequencing and data analysis for BE

To initiate the BE process, *E. coli DH5α* cells containing BE systems were cultured overnight, diluted 1000 times into LB medium containing appropriate antibiotics and 200 μM IPTG, and incubated at 37 °C with continuous shaking for 36 h. After incubation, bacterial cells were collected to extract plasmids using a Plasmid Extraction Kit (Tiangen Biotech, Beijing, China). Genomic DNA from the bacterial cells was extracted with a Bacterial Genome DNA Extraction Kit (Tiangen Biotech, Beijing, China). The targeted sequences were amplified with specific primers, which were designed at ±100 bp surrounding the target sites. The resulting amplicons with both forward and reverse barcodes were purified using an EasyPure PCR Purification Kit (TransGen Biotech, Beijing, China) and quantified with a NanoDrop 2000 spectrophotometer (Thermo Fisher Scientific, Waltham, MA, USA). Equal quantities of the PCR products were pooled, and samples were sequenced by MyGenostics (Beijing, China) using the Illumina NextSeq 500 platform. The crRNA target sites in the sequenced reads were examined for C-to-T substitutions and indels. All experiments were repeated three times to obtain the means and standard variations. Analyses of BE processivity were performed as described previously [13].

## 3 Results

### 3.1 Directed evolution of DNase-inactive Cpf1 for expanded PAM recognition

**Fig. 1:**
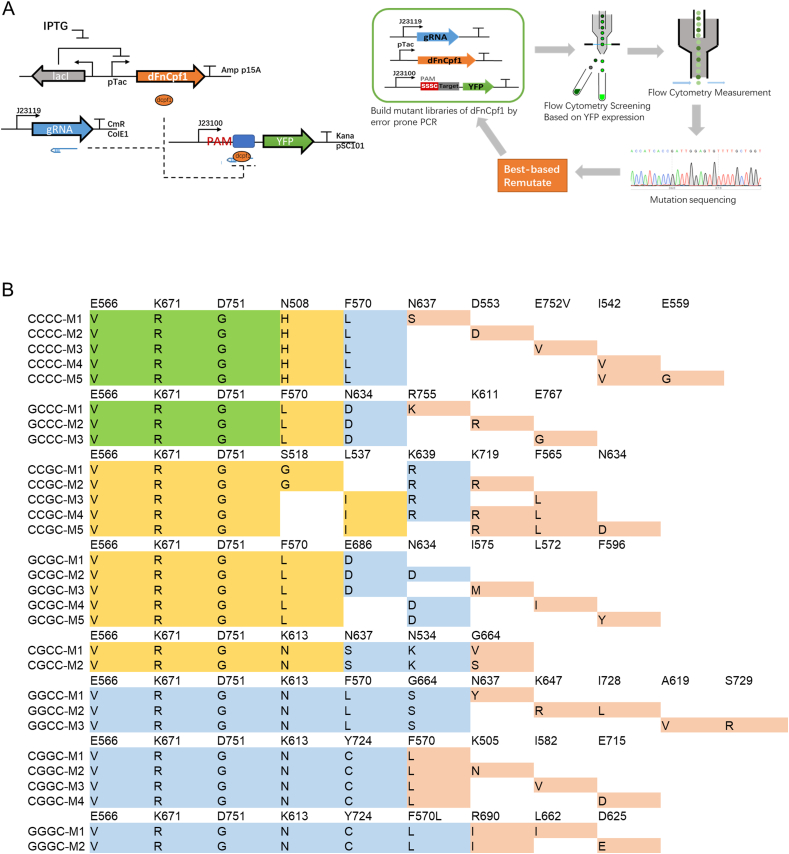
The directed evolution of CRISPR nuclease dFnCpf1. (A) Flow chart of the directed evolution of CRISPR nuclease dFnCpf1. In the negative screening circuit, dFnCpf1 is expressed from an inducible promoter (pTac), crRNA is expressed from a constitutive promoter (J23119), and a reporter gene *yfp* is repressed by the dFnCpf1-crRNA complex in the upstream region of its initial transcription. (B)Variants screened from the fourth round of evolution. Mutations arising from first (green), second (yellow), third (blue), and fourth (orange) round of evolution on eight SSSC PAM sequences were labeled separately. M1 represents the variant with the highest activity in the corresponding PAM trajectories. (Supplementary Table 3).

With an aim to relax PAM constraints and generate new PAM mutant variants, we analyzed the PAM preference of previously reported *Francisella novicida* Cpf1 (FnCpf1). We concluded that FnCpf1 had a preference for PAM TTTV sites rather than TTV. This indicated the -4 T (-1 bit is the last base of the PAM sequence) preference in the PAM sequence [26], [27]. To link Cpf1 PAM recognition to fluorescence value, we developed a bacterial negative screening assay where a constitutively expressed *yfp* gene was targeted in the upstream region of its initial transcription by a crRNA. Expression of the dFnCpf1-crRNA system was induced using IPTG, and reduction in fluorescence was quantified to evaluate the PAM recognition and target binding efficiency of the dFnCpf1-crRNA complex (Fig. 1A). In AsCpf1 with altered PAM specificity, mutated R542 and R607 residues form new interactions with non-thymine nucleotides at the -2 and -3 PAM positions [34]. Using the negative screening assay, we validated that the corresponding dFnCpf1 mutant (N607R/K671R) created by homologous alignment had a low activity on most expected high-GC PAM sites, although it exhibited lower PAM constrains than WT dFnCpf1 (Fig. S1). It suggested that re-engineering PAM specificity might require additional mutations. Therefore, we focused our evolution efforts on the SSSC (S = G, C) subset of PAM sequence space, which is largely inaccessible by commonly used Cpf1 variants. Directed evolution was employed to evolve the dFnCpf1 for targeting each of the eight SSSC PAM sequences in parallel.

Initially, we randomly mutagenized a 789 bp DNA sequence containing the PAM-interacting (PI) domains of dFnCpf1 mutants N607R, K671R, N607R/K671R through error-prone PCR to construct a *dfncpf1* variants plasmid library (Supplementary Table 1). The mutagenesis library was introduced into host *E. coli* cells harboring a plasmid expressing crRNA and a plasmid carrying different SSSC PAMs upstream of the *yfp* gene. Then, we utilized the negative screening selection assay to screen bacterial colonies showing the most significant decrease in fluorescence value, and the mutant regions were sequenced for the next round of mutant screening (Fig. 1A).

Apart from K671R, E566V and D751G mutations (referred to as VRG) were yielded from CCCC and GCCC PAM trajectories in the first round of evolution (Fig. 1B), speculating that a combination of these mutations might permit efficient binding of sites containing GC-rich PAMs. Thus, the VRG mutant was again mutagenized, and the next round of selection was performed against SSSC PAM target sites. In addition to VRG, we obtained another important mutation, K613 N (referred to as VRGN), in the CGCC PAM trajectory (Fig. 1B) to enable efficient recognition of PAMs containing a G at the -3 position. Thus, we mutagenized the VRGN mutant to perform subsequent evolutions against SGSS PAM target sites. Further evolutions were performed to introduce additional mutations across the eight apparent trajectories, respectively. After two subsequent rounds of directed evolutions on each of the eight SSSC PAMs in parallel, we observed distinct sets of mutations depending on the -3 base of the PAM targeted for evolution (Fig. 1B). Eight M1 variants were selected for further characterization as they demonstrated the highest activity in the corresponding PAM trajectories (Fig. 1B, Supplementary Table 3).

### 3.2 PAM preference profiles for wild-type dFnCpf1 and eight effective mutants

**Fig. 2:**
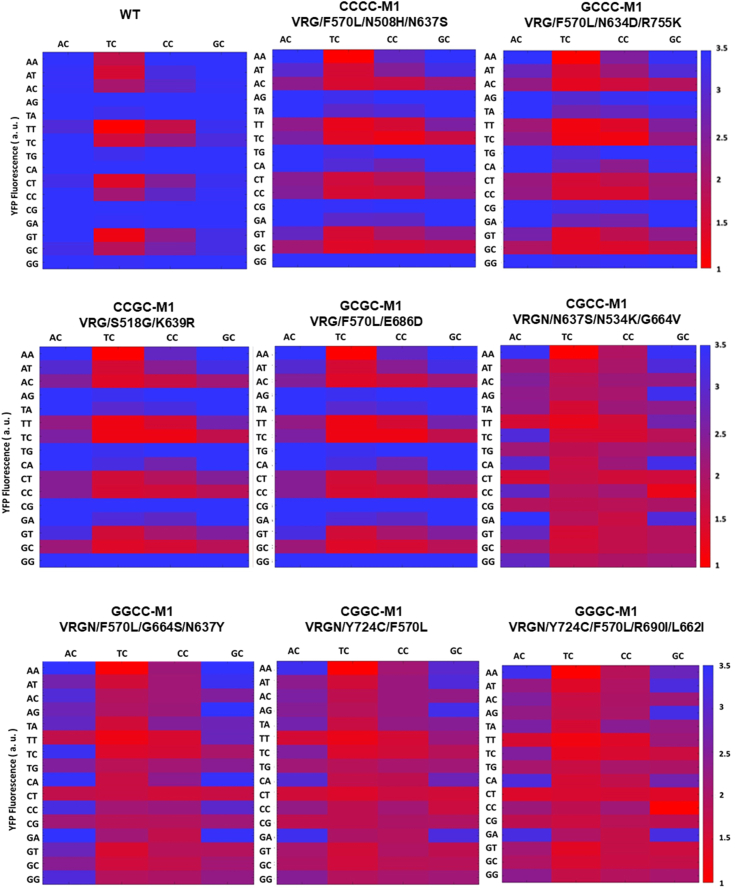
PAM preference profiles for WT dFnCpf1 and eight SSSC-M1 mutants. The most effective mutants, SSSC-M1s, were selected to assess the global 64 PAM (NNNC) preferences using the negative screening assay and later compared with WT dFnCpf1. The mutants CCCC-M1, GCCC-M1, CCGC-M1, and GCGC-M1 were selected from mutants obtained by directed evolution, which recognized CCCC, GCCC, CCGC, and GCGC (SCSC) PAMs respectively. The mutants CGCC-M1, GGCC-M1, CGGC-M1, and GGGC-M1 were selected from mutants obtained by directed evolution, which recognized CGCC, GGCC, CGGC, and GGGC (SGSC) PAMs respectively. YFP fluorescence intensity after induction was used as the characterization value of PAM preference profiles.

To determine PAM compatibilities in acquired mutants (separately evolved based on different GC-rich PAMs), eight M1 variants were assessed for the global 64 PAM preferences (NNNC, the last base of PAM sequence is determined as C due to the weak preference) and later compared with WT dFnCpf1. As expected, WT dFnCpf1 was found to be the most effective for NTTC PAMs, especially for the TTTC PAM. WT dFnCpf1 also targeted other PAM sequences, including NCTC, AATC, and TTCC, but at lower rates (Fig. 2, Supplementary Table 4). By contrast, apart from AATC and NTTC PAMs, the SCSC-M1s variants (CCCC-M1, GCCC-M1, CCGC-M1, GCGC-M1) showed the highest activity at NCCC and NCTC PAMs, compared to little or no activity for WT (Fig. 2). The PAM preferences of SCSC-M1s, which could also recognize NTCC PAMs (and, to a lesser extent, NCGC), were also not as strictly defined as that of WT. Surprisingly, the SGSC-M1s variants (CGCC-M1, GGCC-M1, CGGC-M1, GGGC-M1) were active at almost all 64 PAMs, especially the GC-rich PAMs, although the protein activity declined (Fig. 2, Supplementary Table 4). We observed that the mutant GGGC-M1 (VRGN/Y724C/F570L/R690I/L662I), which was selected from the variants that could recognize GGGC PAM, had 52 effective identification tags (YFP fluorescence value < 200) out of all 64 NNNC sites (81.3%) (Supplementary Table 4). Furthermore, the GGGC-M1 mutant could effectively recognize 28 PAMs out of the 32 (87.5%) GC-rich PAMs (two or more C/G within -2 to -4 bits of PAM sequence) (Supplementary Table 4). Thus, based on these outcomes, the dFnCpf1 (VRGN/Y724C/F570L/R690I/L662I) variant, obtained in this study with a significantly expanded targeting range, was referred to as broad-spectrum dFnCpf1 (bsdFnCpf1).

### 3.3 BE with bsdFnCpf1-cytidine deaminase fusions in *E. coli*

**Fig. 3:**
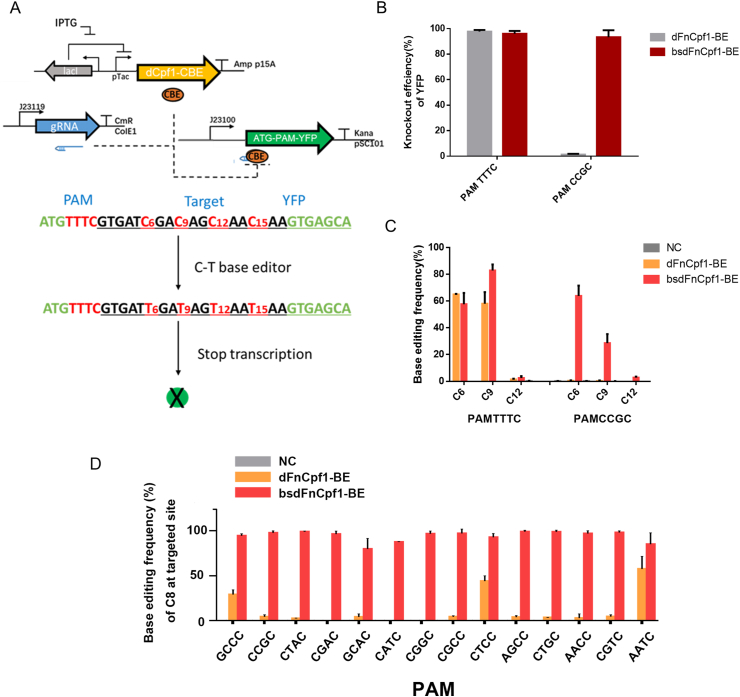
Base editing mediated by bsdFnCpf1-BE and dFnCpf1-BE in *E. coli.* (A) Schematic representation of the YFP-iSTOP reporter system to examine C to T conversion efficiency in *E. coli*. This system contained a target sequence “CGA CAG CAA CAA” after the initiation codon “ATG” of the YFP coding sequence, and successful base editing of C to T resulted in YFP transcription termination. (B) Analysis of the YFP shutdown efficiency using the YFP-iSTOP reporter system. The bar graph displayed the YFP shutdown efficiency of bsdFnCpf1-BE or dFnCpf1-BE after 36 h of IPTG induction. YFP shutdown efficiency was calculated as the percentage of YFP knockout cells among the YFP positive cells in the control group (Fig. S2). (C) Determination of bsdFnCpf1-BE or dFnCpf1-BE induced base-editing frequency at every single cytosine in the indicated “CGA CAG CAA CAA” region. The cytosines were counted with the base proximal to the PAM as position 1. (D) Determination of bsdFnCpf1-BE or dFnCpf1-BE induced base-editing frequency at the target site “GGGCACTCTCCAGATAGGGAT” with different PAMs. Comparison of C to T editing efficiency of C8 at the target site between bsdFnCpf1-BE and dFnCpf1-BE.

Expanding the targeting scope of base editing is a major motivation behind the development of dCpf1 variants with diversified PAM compatibilities. With a broad-spectrum dFnCpf1 generated in this study, we further assessed bsdFnCpf1 based BE system for inducing targeted C to T substitutions in *E. coli*. To determine whether the enhanced activities of bsdFnCpf1 could enable efficient BE, rat APOBEC1 was fused either with dFnCpf1 or bsdFnCpf1, along with uracil DNA glycosylase inhibitor (UGI). This resulted in two dCpf1-based BEs: dFnCpf1-BE and bsdFnCpf1-BE [35]. By effectively converting C into T at three potentially inducible stop (iSTOP) codons, BE of CAG (Gln), CGA (Arg), or CAA (Gln) could introduce TAG, TGA, or TAA stop codons to halt the gene expression. Therefore, to estimate the BE efficiency, the YFP-iSTOP reporter system containing a target sequence fragment including “CGA CAG CAA CAA” codons at positions 6–15, immediately after the initiation codon “ATG” of the YFP coding sequence was constructed. Successful BE of this system was indicated by the termination of YFP expression (Fig. 3A) [36].

We initially analyzed the potential of the constructed dCpf1-based BEs in *E. coli* with the targeted PAM TTTC and CCGC. The base-edited colonies were cultured in LB medium and analyzed by flow cytometry and sequencing. Consistent with the PAM preferences, the YFP shutdown efficiencies for bsdFnCpf1-BE and dFnCpf1-BE were similar in PAM TTTC vector transformed cells (97.7% vs. 95.9%), whereas the editing efficiency was 85-fold higher for bsdFnCpf1-BE in PAM CCGC vector transformed cells than dFnCpf1-BE (93.3% vs. 1.1%) (Fig. 3B). The Sanger sequencing confirmed that inducible stop codons caused YFP knockout. The results suggested that both dFnCpf1-BE and bsdFnCpf1-BE induced stop codons with the PAM TTTC as the target, but only bsdFnCpf1-BE induced C-to-T conversion with PAM CCGC as the target. Moreover, different C-to-T editing frequencies at different positions existed, and the major editing sites were C6 and C9 (Fig. 3C). The APOBEC1-fused BE system showed a strong preference for TC sequences, but almost no editing activity for GC sequences, which lead to low base-editing efficiency for C12. The C-to-T conversion efficiency also rest in the position of C in spacer, C15 is probably not within the editing window of the BE system. The editing positions of BE system were far away from the cleavage site of Cpf1 nuclease, which is due to the different locations of deaminase domain and nuclease domain (Fig. S4). We further analyzed the BE frequency at target site “GGGCACTCTCCAGATAGGGAT” on the reporter plasmids with other non-canonical PAMs. As expected, bsdFnCpf1-BE exhibited substantially improved C to T editing efficiency across all the 14 PAMs (Fig. 3D), in line with previously tested PAM preference profiles (Fig. 2, Supplementary Table 4).

**Fig. 4:**
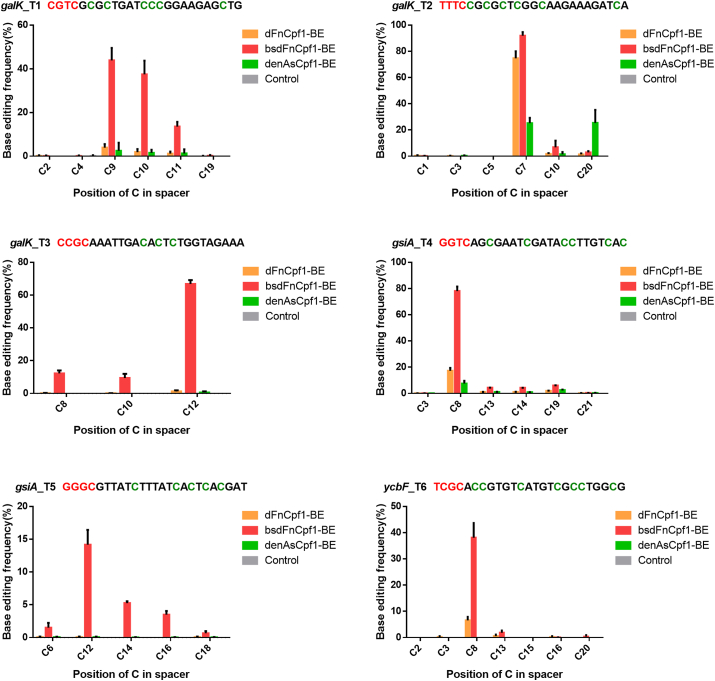
C to T conversion efficiencies mediated by dCpf1-BE across six target sites with different PAM sequences in the *E. coli* genome, assessed by deep sequencing. Comparison of base editing efficiencies mediated by dFnCpf1-BE, bsdFnCpf1-BE, and denAsCpf1-BE across six target sites in the *E. coli* genome. The base editing frequencies were displayed as mean ± SD for three independent experiments. All PAM sequences were in red, and all Cs were highlighted in green for each target site.

Furthermore, we employed the base editor to edit the *E. coli* genome and compared the multiplex editing activities of bsdFnCpf1-BE, dFnCpf1-BE, and recently reported denAsCpf1-BE, targeting genes with six different PAMs [24]. For multiplex editing, tandem repeats of crRNA-expression units were assembled onto a separate plasmid from the one expressing dCpf1-BE proteins. The plasmid targeting six sites in *galK, ycbF,* and *gsiA* was co-introduced into cells with the plasmid expressing bsdFnCpf1-BE, dFnCpf1-BE, or denAsCpf1-BE. The transformants were cultured in LB medium and later induced by IPTG. Genomic DNA was extracted from these bacterial cells, and the target regions were amplified and sequenced. In line with the PAM preference, denAsCpf1-BE and dFnCpf1-BE effectively mutated the target sites bearing PAM TTTC, but only bsdFnCpf1-BE efficiently mutated all of the targeted sites bearing different PAMs simultaneously, with an editing efficiency of up to 85% for the six target sites (Fig. 4).

We showed that the new editing system bsdFnCpf1-BE could achieve efficient multiplex base editing in plasmid and genome. These data validated the greatly improved PAM recognition range of bsdFnCpf1, which enabled bsdFnCpf1 to target more gene sequences. Besides, it could be modified into base editors to achieve efficient multiplex BE. It suggests that the bsdFnCpf1-based editor may be of great potential substitute for the dcas9-based editor, as it is an efficient, multiplex BE technology in *E. coli* and other bacterial species, which have been reported to be suitable for Cpf1.

## 4 Discussion

A key challenge in broadening the application scope of the CRISPR-Cas system is extending the targeting range of genome-editing agents. In the CRISPR-based gene regulation or genome editing system, recognition of the PAM region by nuclease proteins is the cardinal requirement, restricting the target range and flexibility of the CRISPR system. Multiple improved Cpf1 variants (RR, RVR, and enAsCpf1) were constructed to expand the PAM compatibility using structure-guided mutagenesis [23,24]. From a practical perspective, these semi-rational approaches generated small libraries and required relatively little experimental effort to identify moderately altered variants. However, changes in residues far away from the active site might also have a pronounced effect on enzyme activity. These potential beneficial mutations were excluded in these gene-editing campaigns.

**Fig. 5:**
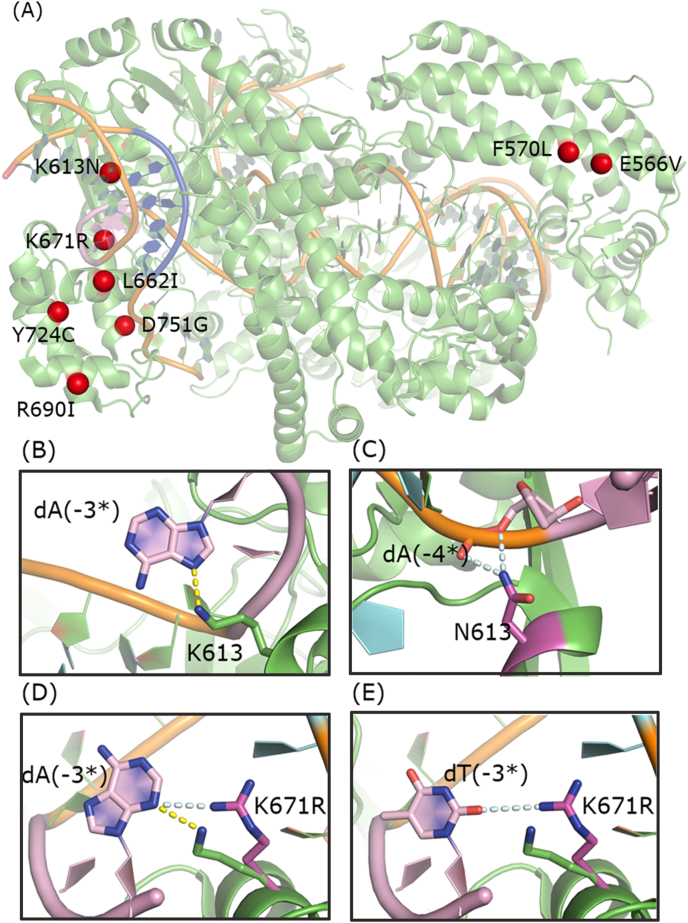
Structural differences between WT dFnCpf1 and bsdFnCpf1. (A) Overall structural model of bsdFnCpf1. Mutated amino acids are marked as dark red spheres. PAM nucleotides are marked as blue. (B) Detailed view of K613 interaction with nucleotide dA complementary to PAM. (C) Detailed view of N613 interaction with the phosphate backbone of nucleotides complementary to PAM. (D) Structural differences in K671 (K671R) interacting with PAM-complementary nucleotide between WT and bsdFnCpf1 **(PAM TTTC)**. (E) Structural differences in K671 (K671R) interacting with PAM-complementary nucleotide between WT and bsdFnCpf1 **(PAM TATC)**. The substituted residues are highlighted by purple labels. The structural figures were prepared using PYMOL (http://pymol.org).

Thus, in this study, we developed a fluorescence-based high-throughput screening system to access Cpf1's unexplored regions of sequence space. The optimized mutant bsFnCpf1 contains eight mutations, which are scattered throughout the protein (Fig. 5A). The K671R and K613 N mutations in proximity to the PAM sequence in the WT FnCpf1 structure are likely to affect the PAM recognition directly [23]. In WT structure model, K671 contacts the C2 carbonyl in dT (-2), the N3 of dA (-3*) in the PAM sequence and the oxygen of the deoxyribose of dA (-4*), whereas K671R may confer much stronger hydrogen bond interactions with dA (-3*) (Fig. 5D). Moreover, the guanidine group of K671R may form much stronger hydrogen bond interactions with dT (-3*) and dG (-3*) (Fig. 5E). Remarkably, we found that the K613N mutation may form two hydrogen bonds with the phosphate backbone of nucleotides complementary to PAM instead of base-specific interactions, thereby achieving the relaxed PAM recognition (Fig. 5B and C). Apart from these two mutations, random mutations were accumulated during the irrational evolution process. These mutations occur at sites distant from the PAM-containing DNA duplexes, which might undergo subtle structural changes. The structural model shows that the last base pair in the PAM duplex does not form base-specific contacts with the protein, which confirmed that PAM strength is not sensitive to the last position. Together, these alterations could result in better PAM recognition efficiency than previous Cpf1 variants with limited mutagenesis.

In conclusion, bsdFnCpf1 engineered in this study efficiently targeted previously inaccessible multiple PAMs. Owing to its simple composition and universality, we envision that bsdFnCpf1 could be a powerful tool for synthetic biology, which may aid metabolic pathway regulation or BE in engineered hosts.

## Declaration of competing interest

The authors declare that they have no known competing financial interests or personal relationships that could have appeared to influence the work reported in this paper.

## Credit author statement

Zehua Chen: Investigation, Methodology, Writing – original draft, Jinyuan Sun: Visualization, Formal analysis, Ying Guan: Investigation, Ming Li: Investigation, Chunbo Lou: Conceptualization, Supervision, Bian Wu: Funding acquisition, Supervision, Writing – review & editing
