# Supplementary material for: Engineered DNase-inactive Cpf1 variants to improve targeting scope for base editing in E. coli
Source: Synth Syst Biotechnol. 2021 Sep 24;6(4):326–34. doi: 10.1016/j.synbio.2021.09.002 (PMC8484740; doi:10.1016/j.synbio.2021.09.002)
Supplement: Multimedia component 1 [file mmc1.docx]

**Engineered** **DNase-inactive Cpf1 Variants to Improve Targeting Scope for Base Editing in *E. coli***

Zehua Chen ^a, b, 1^, Jinyuan Sun ^a, b^, Ying Guan ^c^, Ming Li ^a^, Chunbo Lou ^a*^, Bian Wu ^a*^

^a^ CAS Key Laboratory of Microbial Physiological & Metabolic Engineering and State Key Laboratory of Microbial Resources, Institute of Microbiology, Chinese Academy of Sciences, Beijing, 100101, China

^b^ College of Life Sciences, University of Chinese Academy of Sciences, Beijing, 100149, China

^c^ Tsinghua University, Beijing 100084, China

* Correspondence may also be addressed to Chunbo Lou (Email: louchunbo@gmail.com) or Bian Wu (wub@im.ac.cn)


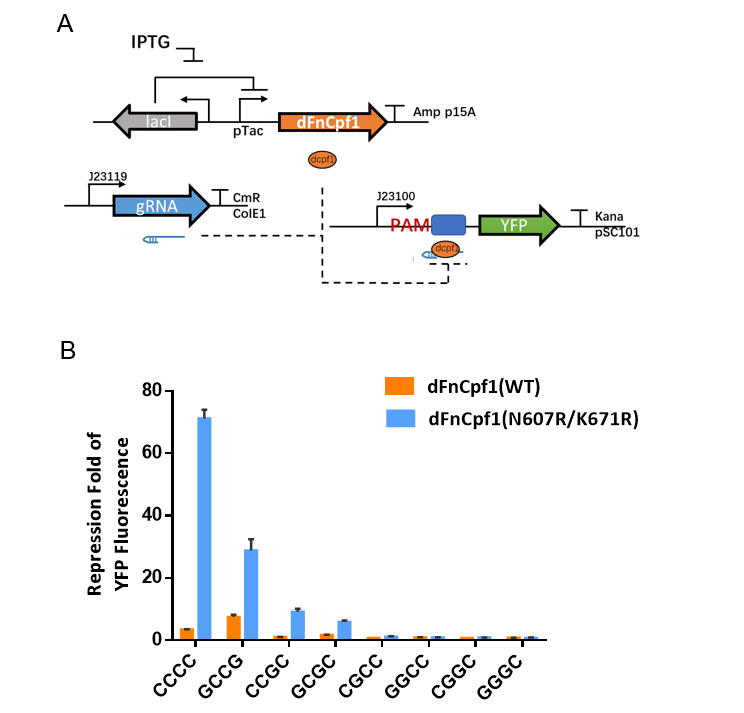


**Figure S1. Activities of N607R/K671R mutant and wild-type dFnCpf1 binding to sites with PAM SSSC using the negative screening assay.** (**A**) Schematic representation of the negative screening assay. (**B**) Repression fold of fluorescence intensity caused by wild-type dFnCpf1 and the mutant dFnCpf1 (N607R/K671R) in recognition of PAM SSSC (S=C, G).


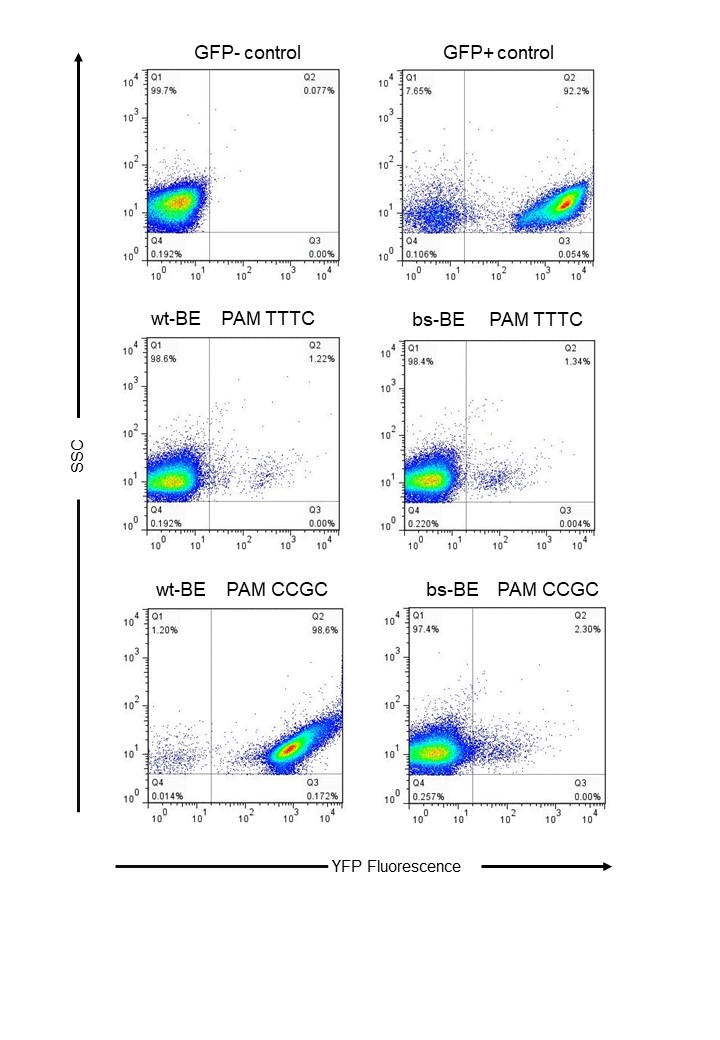


**Figure S2.** **Flow cytometry analysis of base-editing efficiency based on YFP fluorescence in the YFP-iSTOP reporter system.** Flow cytometry analysis of YFP shutdown by dFnCpf1-BE (wt-BE) or bsdFnCpf1-BE (bs-BE) in the PAM TTTC and CCGC vector. The morphological of bacteria was measured by side scatter light (SSC). The fluorescence of the *E. coli* cells was quantified. The selected region of low-YFP cells were counted to determine knockouts. YFP shutdown efficiency was calculated as the percentage of YFP knockout cells among the YFP-positive cells in the control group. These data showed a representative experiment from three independent experiments.


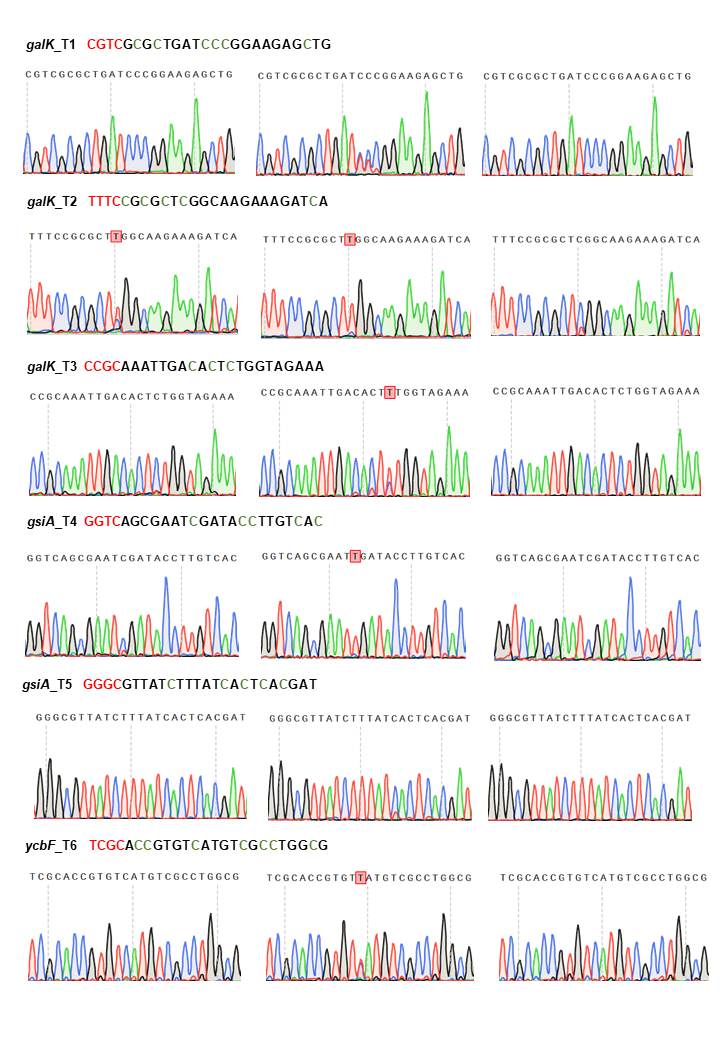


**Figure S3. Sanger sequencing of base-editing at six different loci in the *glaK*, *gsiA*, *ycbF* gene of *E. coli*.** The modified genomes were extracted from bacterial populations in each of the experimental groups and the target regions were amplified and sequenced. PAM motifs are in red and all Cs in the target sites are in green. The transformations and base-editing assays were repeated for three times. The data showed a peak diagram of sequencing of the targeted loci in the base-editing assays for dFnCpf1-BE, bsdFnCpf1-BE, and denAsCpf1-BE from left to right.


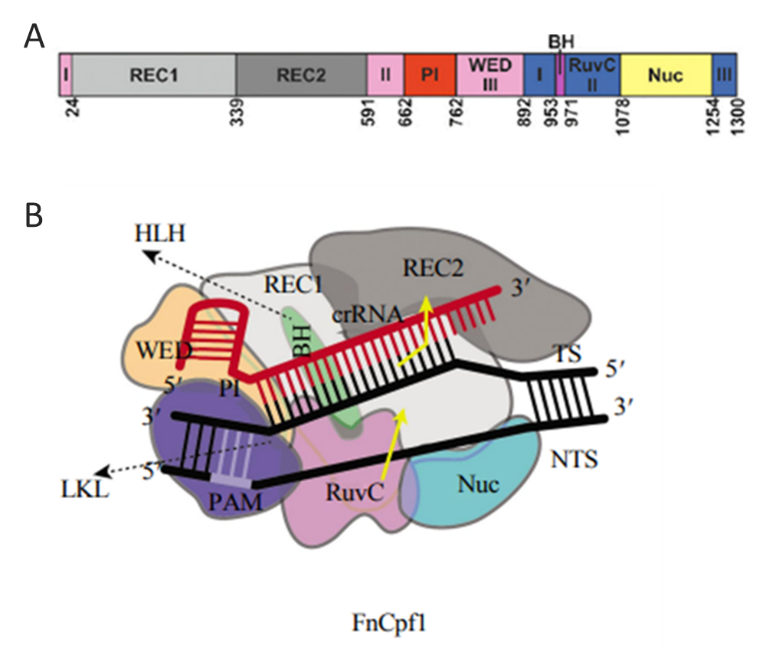


**Figure S4. Structure of the FnCpf1-crRNA-target DNA complex.** (A) Domain organization of FnCpf1. (B) Schematic of FnCpf1 in complex with the crRNA and its target DNA. DNA nuclease reaction takes place in a pocket at the interface between the RuvC and Nuc domains, in which the E1006 and R1218 residues have an important role in catalysis near the C terminal. Accordingly, The RuvC and Nuc domains should be located at positions suitable to induce staggered DNA double-strand breaks at the PAM-distal positions. For the modified dCpf1-BE, dCpf1 only retains DNA-targeting ability after binding to a guide RNA, while the N-terminus fused deaminase is more likely to catalyze the bases closer to the PAM, which may explain the variation in editing positions.

**Supplementary Table 1. Example sequences of promoters, RBS and representative genes used in this study are included below.**

**1.pTac-dFnCpf1**

pTac promoter in bold blue, RiboJ insulators in green, RBS in orange, dFnCpf1 in black with the targeted mutation region underlined

TGTTGACAATTAATCATCGGCTCGTATAATGTGTGGAATTGTGAGCGCTCACAATTAGCTGTCACCGGATGTGCTTTCCGGTCTGATGAGTCCGTGAGGACGAAACAGCCTCTACAAATAATTTTGTTTAAAAAGAGGAGAAAATGTCAATTTATCAAGAATTTGTTAATAAATATAGTTTAAGTAAAACTCTAAGATTTGAGTTAATCCCACAGGGTAAAACACTTGAAAACATAAAAGCAAGAGGTTTGATTTTAGATGATGAGAAAAGAGCTAAAGACTACAAAAAGGCTAAACAAATAATTGATAAATATCATCAGTTTTTTATAGAGGAGATATTAAGTTCGGTTTGTATTAGCGAAGATTTATTACAAAACTATTCTGATGTTTATTTTAAACTTAAAAAGAGTGATGATGATAATCTACAAAAAGATTTTAAAAGTGCAAAAGATACGATAAAGAAACAAATATCTGAATATATAAAGGACTCAGAGAAATTTAAGAATTTGTTTAATCAAAACCTTATCGATGCTAAAAAAGGGCAAGAGTCAGATTTAATTCTATGGCTAAAGCAATCTAAGGATAATGGTATAGAACTATTTAAAGCCAATAGTGATATCACAGATATAGATGAGGCGTTAGAAATAATCAAATCTTTTAAAGGTTGGACAACTTATTTTAAGGGTTTTCATGAAAATAGAAAAAATGTTTATAGTAGCAATGATATTCCTACATCTATTATTTATAGGATAGTAGATGATAATTTGCCTAAATTTCTAGAAAATAAAGCTAAGTATGAGAGTTTAAAAGACAAAGCTCCAGAAGCTATAAACTATGAACAAATTAAAAAAGATTTGGCAGAAGAGCTAACCTTTGATATTGACTACAAAACATCTGAAGTTAATCAAAGAGTTTTTTCACTTGATGAAGTTTTTGAGATAGCAAACTTTAATAATTATCTAAATCAAAGTGGTATTACTAAATTTAATACTATTATTGGTGGTAAATTTGTAAATGGTGAAAATACAAAGAGAAAAGGTATAAATGAATATATAAATCTATACTCACAGCAAATAAATGATAAAACACTCAAAAAATATAAAATGAGTGTTTTATTTAAGCAAATTTTAAGTGATACAGAATCTAAATCTTTTGTAATTGATAAGTTAGAAGATGATAGTGATGTAGTTACAACGATGCAAAGTTTTTATGAGCAAATAGCAGCTTTTAAAACAGTAGAAGAAAAATCTATTAAAGAAACACTATCTTTATTATTTGATGATTTAAAAGCTCAAAAACTTGATTTGAGTAAAATTTATTTTAAAAATGATAAATCTCTTACTGATCTATCACAACAAGTTTTTGATGATTATAGTGTTATTGGTACAGCGGTACTAGAATATATAACTCAACAAATAGCACCTAAAAATCTTGATAACCCTAGTAAGAAAGAGCAAGAATTAATAGCCAAAAAAACTGAAAAAGCAAAATACTTATCTCTAGAAACTATAAAGCTTGCCTTAGAAGAATTTAATAAGCATAGAGATATAGATAAACAGTGTAGGTTTGAAGAAATACTTGCAAACTTTGCGGCTATTCCGATGATATTTGATGAAATAGCTCAAAACAAAGACAATTTGGCACAGATATCTATCAAATATCAAAATCAAGGTAAAAAAGACCTACTTCAAGCTAGTGCGGAAGATGATGTTAAAGCTATCAAGGATCTTTTAGATCAAACTAATAATCTCTTACATAAACTAAAAATATTTCATATTAGTCAGTCAGAAGATAAGGCAAATATTTTAGACAAGGATGAGCATTTTTATCTAGTATTTGAGGAGTGCTACTTTGAGCTAGCGAATATAGTGCCTCTTTATAACAAAATTAGAAACTATATAACTCAAAAGCCATATAGTGATGAGAAATTTAAGCTCAATTTTGAGAACTCGACTTTGGCTAATGGTTGGGATAAAAATAAAGAGCCTGACAATACGGCAATTTTATTTATCAAAGATGATAAATATTATCTGGGTGTGATGAATAAGAAAAATAACAAAATATTTGATGATAAAGCTATCAAAGAAAATAAAGGCGAGGGTTATAAAAAAATTGTTTATAAACTTTTACCTGGCGCAAATAAAATGTTACCTAAGGTTTTCTTTTCTGCTAAATCTATAAAATTTTATAATCCTAGTGAAGATATACTTAGAATAAGAAATCATTCCACACATACAAAAAATGGTAGTCCTCAAAAAGGATATGAAAAATTTGAGTTTAATATTGAAGATTGCCGAAAATTTATAGATTTTTATAAACAGTCTATAAGTAAGCATCCGGAGTGGAAAGATTTTGGATTTAGATTTTCTGATACTCAAAGATATAATTCTATAGATGAATTTTATAGAGAAGTTGAAAATCAAGGCTACAAACTAACTTTTGAAAATATATCAGAGAGCTATATTGATAGCGTAGTTAATCAGGGTAAATTGTACCTATTCCAAATCTATAATAAAGATTTTTCAGCTTATAGCAAAGGGCGACCAAATCTACATACTTTATATTGGAAAGCGCTGTTTGATGAGAGAAATCTTCAAGATGTGGTTTATAAGCTAAATGGTGAGGCAGAGCTTTTTTATCGTAAACAATCAATACCTAAAAAAATCACTCACCCAGCTAAAGAGGCAATAGCTAATAAAAACAAAGATAATCCTAAAAAAGAGAGTGTTTTTGAATATGATTTAATCAAAGATAAACGCTTTACTGAAGATAAGTTTTTCTTTCACTGTCCTATTACAATCAATTTTAAATCTAGTGGAGCTAATAAGTTTAATGATGAAATCAATTTATTGCTAAAAGAAAAAGCAAATGATGTTCATATATTAAGTATAGCAAGAGGTGAAAGACATTTAGCTTACTATACTTTGGTAGATGGTAAAGGCAATATCATCAAACAAGATACTTTCAACATCATTGGTAATGATAGAATGAAAACAAACTACCATGATAAGCTTGCTGCAATAGAGAAAGATAGGGATTCAGCTAGGAAAGACTGGAAAAAGATAAATAACATCAAAGAGATGAAAGAGGGCTATCTATCTCAGGTAGTTCATGAAATAGCTAAGCTAGTTATAGAGTATAATGCTATTGTGGTTTTTGAGGATTTAAATTTTGGATTTAAAAGAGGGCGTTTCAAGGTAGAGAAGCAGGTCTATCAAAAGTTAGAAAAAATGCTAATTGAGAAACTAAACTATCTAGTTTTCAAAGATAATGAGTTTGATAAAACTGGGGGAGTGCTTAGAGCTTATCAGCTAACAGCACCTTTTGAGACTTTTAAAAAGATGGGTAAACAAACAGGTATTATCTACTATGTACCAGCTGGTTTTACTTCAAAAATTTGTCCTGTAACTGGTTTTGTAAATCAGTTATATCCTAAGTATGAAAGTGTCAGCAAATCTCAAGAGTTCTTTAGTAAGTTTGACAAGATTTGTTATAACCTTGATAAGGGCTATTTTGAGTTTAGTTTTGATTATAAAAACTTTGGTGACAAGGCTGCCAAAGGCAAGTGGACTATAGCTAGCTTTGGGAGTAGATTGATTAACTTTAGAAATTCAGATAAAAATCATAATTGGGATACTCGAGAAGTTTATCCAACTAAAGAGTTGGAGAAATTGCTAAAAGATTATTCTATCGAATATGGGCATGGCGAATGTATCAAAGCAGCTATTTGCGGTGAGAGCGACAAAAAGTTTTTTGCTAAGCTAACTAGTGTCCTAAATACTATCTTACAAATGCGTAACTCAAAAACAGGTACTGAGTTAGATTATCTAATTTCACCAGTAGCAGATGTAAATGGCAATTTCTTTGATTCGCGACAGGCGCCAAAAAATATGCCTCAAGATGCTGATGCCAATGGTGCTTATCATATTGGGCTAAAAGGTCTGATGCTACTAGGTAGGATCAAAAATAATCAAGAGGGCAAAAAACTCAATTTGGTTATCAAAAATGAAGAGTATTTTGAGTTCGTGCAGAATAGGAATAACTAG

**2. J23119- FnCpf1-crRNA**

J23119 promoter in bold blue, full repeat of FnCpf1 crRNA in orange, spacer sequence in red, terminator in black

TTGACAGCTAGCTCAGTCCTAGGTATAATACTAGTGTCTAAGAACTTTAAATAATTTCTACTGTTGTAGATCGTGCGTGGCGAGGGTGAAGGGTCTAAGAACTTTAAATAATTTCTACTGTTGTAGATGAAGCTTGGGCCCGAACAAAAACTCATCTCAGAAGAGGATCTGAATAGCGCCGTCGACCATCATCATCATCATCATTGAGTTTAAACGGTCTCCAGCTTGGCTGTTTTGGCGGATGAGAGAAGATTTTCAGCCTGATACAGATTAAATCAGAACGCAGAAGCGGTCTGATAAAACAGAATTTGCCTGGCGGCAGTAGCGCGGTGGTCCCACCTGACCCCATGCCGAACTCAGAAGTGAAACGCCGTAGCGCCGATGGTAGTGTGGGGTCTCCCCATGCGAGAGTAGGGAACTGCCAGGCATCAAATAAAACGAAAGGCTCAGTCGAAAGACTGGGCCTTTCGTTTTATCTGTTGTTTGTCGGTGAACT

**3. J23100-PAM-Target-YFP**

J23100 promoter in bold blue, PAM underlined in purple, target sites in red, RBS in orange, YFP in green

TTGACGGCTAGCTCAGTCCTAGGTACAGTGCTAGCATCAGCAGGACGCACTGACCACTGGATGGTGATGTCAACGGTCATAATGSSSCCGTGCGTGGCGAGGGTGAAGGTGACGCAACTAATGGTTAAAAGAGGAGAAAATGGTGAGCAAGGGCGAGGAGCTGTTCACCGGGGTGGTGCCCATCCTGGTCGAGCTGGACGGCGACGTAAACGGCCACAAGTTCAGCGTGTCCGGCGAGGGCGAGGGCGATGCCACCTACGGCAAGCTGACCCTGAAGTTCATCTGCACCACAGGCAAGCTGCCCGTGCCCTGGCCCACCCTCGTGACCACCTTCGGCTACGGCCTGCAATGCTTCGCCCGCTACCCCGACCACATGAAGCTGCACGACTTCTTCAAGTCCGCCATGCCCGAAGGCTACGTCCAGGAGCGCACCATCTTCTTCAAGGACGACGGCAACTACAAGACCCGCGCCGAGGTGAAGTTCGAGGGCGACACCCTGGTGAACCGCATCGAGCTGAAGGGCATCGACTTCAAGGAGGACGGCAACATCCTGGGGCACAAGCTGGAGTACAACTACAACAGCCACAACGTCTATATCATGGCCGACAAGCAGAAGAACGGCATCAAGGTGAACTTCAAGATCCGCCACAACATCGAGGACGGCAGCGTGCAGCTCGCCGACCACTACCAGCAGAACACCCCCATCGGCGACGGCCCCGTGCTGCTGCCCGACAACCACTACCTGAGCTACCAGTCCGCCCTGAGCAAAGACCCCAACGAGAAGCGCGATCACATGGTCCTGCTGGAGTTCGTGACCGCCGCCGGGATCACTCTCGGCATGGACGAGCTGTACAAGTAA

**4. pTAC-Apobec1-dFnCpf1-UGI**

pTac promoter in bold blue, RiboJ insulators in green, RBS in orange, Apobec1 in bold blue, dFnCpf1 in black, UGI in orange, linker sequences in gray

TGTTGACAATTAATCATCGGCTCGTATAATGTGTGGAATTGTGAGCGCTCACAATTAGCTGTCACCGGATGTGCTTTCCGGTCTGATGAGTCCGTGAGGACGAAACAGCCTCTACAAATAATTTTGTTTAAAAAGAGGAGAAAATGAGCTCAGAGACTGGCCCAGTGGCTGTGGACCCCACATTGAGACGGCGGATCGAGCCCCATGAGTTTGAGGTATTCTTCGATCCGAGAGAGCTCCGCAAGGAGACCTGCCTGCTTTACGAAATTAATTGGGGGGGCCGGCACTCCATTTGGCGACATACATCACAGAACACTAACAAGCACGTCGAAGTCAACTTCATCGAGAAGTTCACGACAGAAAGATATTTCTGTCCGAACACAAGGTGCAGCATTACCTGGTTTCTCAGCTGGAGCCCATGCGGCGAATGTAGTAGGGCCATCACTGAATTCCTGTCAAGGTATCCCCACGTCACTCTGTTTATTTACATCGCAAGGCTGTACCACCACGCTGACCCCCGCAATCGACAAGGCCTGCGGGATTTGATCTCTTCAGGTGTGACTATCCAAATTATGACTGAGCAGGAGTCAGGATACTGCTGGAGAAACTTTGTGAATTATAGCCCGAGTAATGAAGCCCACTGGCCTAGGTATCCCCATCTGTGGGTACGACTGTACGTTCTTGAACTGTACTGCATCATACTGGGCCTGCCTCCTTGTCTCAACATTCTGAGAAGGAAGCAGCCACAGCTGACATTCTTTACCATCGCTCTTCAGTCTTGTCATTACCAGCGACTGCCCCCACACATTCTCTGGGCCACCGGGTTGAAAAGCGGCAGCGAGACTCCCGGGACCTCAGAGTCCGCCACACCCGAAAGTATGTCAATTTATCAAGAATTTGTTAATAAATATAGTTTAAGTAAAACTCTAAGATTTGAGTTAATCCCACAGGGTAAAACACTTGAAAACATAAAAGCAAGAGGTTTGATTTTAGATGATGAGAAAAGAGCTAAAGACTACAAAAAGGCTAAACAAATAATTGATAAATATCATCAGTTTTTTATAGAGGAGATATTAAGTTCGGTTTGTATTAGCGAAGATTTATTACAAAACTATTCTGATGTTTATTTTAAACTTAAAAAGAGTGATGATGATAATCTACAAAAAGATTTTAAAAGTGCAAAAGATACGATAAAGAAACAAATATCTGAATATATAAAGGACTCAGAGAAATTTAAGAATTTGTTTAATCAAAACCTTATCGATGCTAAAAAAGGGCAAGAGTCAGATTTAATTCTATGGCTAAAGCAATCTAAGGATAATGGTATAGAACTATTTAAAGCCAATAGTGATATCACAGATATAGATGAGGCGTTAGAAATAATCAAATCTTTTAAAGGTTGGACAACTTATTTTAAGGGTTTTCATGAAAATAGAAAAAATGTTTATAGTAGCAATGATATTCCTACATCTATTATTTATAGGATAGTAGATGATAATTTGCCTAAATTTCTAGAAAATAAAGCTAAGTATGAGAGTTTAAAAGACAAAGCTCCAGAAGCTATAAACTATGAACAAATTAAAAAAGATTTGGCAGAAGAGCTAACCTTTGATATTGACTACAAAACATCTGAAGTTAATCAAAGAGTTTTTTCACTTGATGAAGTTTTTGAGATAGCAAACTTTAATAATTATCTAAATCAAAGTGGTATTACTAAATTTAATACTATTATTGGTGGTAAATTTGTAAATGGTGAAAATACAAAGAGAAAAGGTATAAATGAATATATAAATCTATACTCACAGCAAATAAATGATAAAACACTCAAAAAATATAAAATGAGTGTTTTATTTAAGCAAATTTTAAGTGATACAGAATCTAAATCTTTTGTAATTGATAAGTTAGAAGATGATAGTGATGTAGTTACAACGATGCAAAGTTTTTATGAGCAAATAGCAGCTTTTAAAACAGTAGAAGAAAAATCTATTAAAGAAACACTATCTTTATTATTTGATGATTTAAAAGCTCAAAAACTTGATTTGAGTAAAATTTATTTTAAAAATGATAAATCTCTTACTGATCTATCACAACAAGTTTTTGATGATTATAGTGTTATTGGTACAGCGGTACTAGAATATATAACTCAACAAATAGCACCTAAAAATCTTGATAACCCTAGTAAGAAAGAGCAAGAATTAATAGCCAAAAAAACTGAAAAAGCAAAATACTTATCTCTAGAAACTATAAAGCTTGCCTTAGAAGAATTTAATAAGCATAGAGATATAGATAAACAGTGTAGGTTTGAAGAAATACTTGCAAACTTTGCGGCTATTCCGATGATATTTGATGAAATAGCTCAAAACAAAGACAATTTGGCACAGATATCTATCAAATATCAAAATCAAGGTAAAAAAGACCTACTTCAAGCTAGTGCGGAAGATGATGTTAAAGCTATCAAGGATCTTTTAGATCAAACTAATAATCTCTTACATAAACTAAAAATATTTCATATTAGTCAGTCAGAAGATAAGGCAAATATTTTAGACAAGGATGAGCATTTTTATCTAGTATTTGAGGAGTGCTACTTTGAGCTAGCGAATATAGTGCCTCTTTATAACAAAATTAGAAACTATATAACTCAAAAGCCATATAGTGATGAGAAATTTAAGCTCAATTTTGAGAACTCGACTTTGGCTAATGGTTGGGATAAAAATAAAGAGCCTGACAATACGGCAATTTTATTTATCAAAGATGATAAATATTATCTGGGTGTGATGAATAAGAAAAATAACAAAATATTTGATGATAAAGCTATCAAAGAAAATAAAGGCGAGGGTTATAAAAAAATTGTTTATAAACTTTTACCTGGCGCAAATAAAATGTTACCTAAGGTTTTCTTTTCTGCTAAATCTATAAAATTTTATAATCCTAGTGAAGATATACTTAGAATAAGAAATCATTCCACACATACAAAAAATGGTAGTCCTCAAAAAGGATATGAAAAATTTGAGTTTAATATTGAAGATTGCCGAAAATTTATAGATTTTTATAAACAGTCTATAAGTAAGCATCCGGAGTGGAAAGATTTTGGATTTAGATTTTCTGATACTCAAAGATATAATTCTATAGATGAATTTTATAGAGAAGTTGAAAATCAAGGCTACAAACTAACTTTTGAAAATATATCAGAGAGCTATATTGATAGCGTAGTTAATCAGGGTAAATTGTACCTATTCCAAATCTATAATAAAGATTTTTCAGCTTATAGCAAAGGGCGACCAAATCTACATACTTTATATTGGAAAGCGCTGTTTGATGAGAGAAATCTTCAAGATGTGGTTTATAAGCTAAATGGTGAGGCAGAGCTTTTTTATCGTAAACAATCAATACCTAAAAAAATCACTCACCCAGCTAAAGAGGCAATAGCTAATAAAAACAAAGATAATCCTAAAAAAGAGAGTGTTTTTGAATATGATTTAATCAAAGATAAACGCTTTACTGAAGATAAGTTTTTCTTTCACTGTCCTATTACAATCAATTTTAAATCTAGTGGAGCTAATAAGTTTAATGATGAAATCAATTTATTGCTAAAAGAAAAAGCAAATGATGTTCATATATTAAGTATAGCAAGAGGTGAAAGACATTTAGCTTACTATACTTTGGTAGATGGTAAAGGCAATATCATCAAACAAGATACTTTCAACATCATTGGTAATGATAGAATGAAAACAAACTACCATGATAAGCTTGCTGCAATAGAGAAAGATAGGGATTCAGCTAGGAAAGACTGGAAAAAGATAAATAACATCAAAGAGATGAAAGAGGGCTATCTATCTCAGGTAGTTCATGAAATAGCTAAGCTAGTTATAGAGTATAATGCTATTGTGGTTTTTGAGGATTTAAATTTTGGATTTAAAAGAGGGCGTTTCAAGGTAGAGAAGCAGGTCTATCAAAAGTTAGAAAAAATGCTAATTGAGAAACTAAACTATCTAGTTTTCAAAGATAATGAGTTTGATAAAACTGGGGGAGTGCTTAGAGCTTATCAGCTAACAGCACCTTTTGAGACTTTTAAAAAGATGGGTAAACAAACAGGTATTATCTACTATGTACCAGCTGGTTTTACTTCAAAAATTTGTCCTGTAACTGGTTTTGTAAATCAGTTATATCCTAAGTATGAAAGTGTCAGCAAATCTCAAGAGTTCTTTAGTAAGTTTGACAAGATTTGTTATAACCTTGATAAGGGCTATTTTGAGTTTAGTTTTGATTATAAAAACTTTGGTGACAAGGCTGCCAAAGGCAAGTGGACTATAGCTAGCTTTGGGAGTAGATTGATTAACTTTAGAAATTCAGATAAAAATCATAATTGGGATACTCGAGAAGTTTATCCAACTAAAGAGTTGGAGAAATTGCTAAAAGATTATTCTATCGAATATGGGCATGGCGAATGTATCAAAGCAGCTATTTGCGGTGAGAGCGACAAAAAGTTTTTTGCTAAGCTAACTAGTGTCCTAAATACTATCTTACAAATGCGTAACTCAAAAACAGGTACTGAGTTAGATTATCTAATTTCACCAGTAGCAGATGTAAATGGCAATTTCTTTGATTCGCGACAGGCGCCAAAAAATATGCCTCAAGATGCTGATGCCAATGGTGCTTATCATATTGGGCTAAAAGGTCTGATGCTACTAGGTAGGATCAAAAATAATCAAGAGGGCAAAAAACTCAATTTGGTTATCAAAAATGAAGAGTATTTTGAGTTCGTGCAGAATAGGAATAACTCTGGTGGTTCTTCTGGTGGTTCTACTAATCTGTCAGATATTATTGAAAAGGAGACCGGTAAGCAACTGGTTATCCAGGAATCCATCCTCATGCTCCCAGAGGAGGTGGAAGAAGTCATTGGGAACAAGCCGGAAAGCGATATACTCGTGCACACCGCCTACGACGAGAGCACCGACGAGAATGTCATGCTTCTGACTAGCGACGCCCCTGAATACAAGCCTTGGGCTCTGGTCATACAGGATAGCAACGGTGAGAACAAGATTAAGATGCTCTAG

**5. pTAC-Apobec1-bsdFnCpf1-UGI**

pTac promoter in bold blue, RiboJ insulators in green, RBS in orange, Apobec1 in bold blue, dFnCpf1 in black, modified codons for bsdFnCpf1 (E566V/K671R/D751G/K613N/Y724C/F570L/R690I/L662I) in red, UGI in orange, linker sequences in gray

TGTTGACAATTAATCATCGGCTCGTATAATGTGTGGAATTGTGAGCGCTCACAATTAGCTGTCACCGGATGTGCTTTCCGGTCTGATGAGTCCGTGAGGACGAAACAGCCTCTACAAATAATTTTGTTTAAAAAGAGGAGAAAATGAGCTCAGAGACTGGCCCAGTGGCTGTGGACCCCACATTGAGACGGCGGATCGAGCCCCATGAGTTTGAGGTATTCTTCGATCCGAGAGAGCTCCGCAAGGAGACCTGCCTGCTTTACGAAATTAATTGGGGGGGCCGGCACTCCATTTGGCGACATACATCACAGAACACTAACAAGCACGTCGAAGTCAACTTCATCGAGAAGTTCACGACAGAAAGATATTTCTGTCCGAACACAAGGTGCAGCATTACCTGGTTTCTCAGCTGGAGCCCATGCGGCGAATGTAGTAGGGCCATCACTGAATTCCTGTCAAGGTATCCCCACGTCACTCTGTTTATTTACATCGCAAGGCTGTACCACCACGCTGACCCCCGCAATCGACAAGGCCTGCGGGATTTGATCTCTTCAGGTGTGACTATCCAAATTATGACTGAGCAGGAGTCAGGATACTGCTGGAGAAACTTTGTGAATTATAGCCCGAGTAATGAAGCCCACTGGCCTAGGTATCCCCATCTGTGGGTACGACTGTACGTTCTTGAACTGTACTGCATCATACTGGGCCTGCCTCCTTGTCTCAACATTCTGAGAAGGAAGCAGCCACAGCTGACATTCTTTACCATCGCTCTTCAGTCTTGTCATTACCAGCGACTGCCCCCACACATTCTCTGGGCCACCGGGTTGAAAAGCGGCAGCGAGACTCCCGGGACCTCAGAGTCCGCCACACCCGAAAGTATGTCAATTTATCAAGAATTTGTTAATAAATATAGTTTAAGTAAAACTCTAAGATTTGAGTTAATCCCACAGGGTAAAACACTTGAAAACATAAAAGCAAGAGGTTTGATTTTAGATGATGAGAAAAGAGCTAAAGACTACAAAAAGGCTAAACAAATAATTGATAAATATCATCAGTTTTTTATAGAGGAGATATTAAGTTCGGTTTGTATTAGCGAAGATTTATTACAAAACTATTCTGATGTTTATTTTAAACTTAAAAAGAGTGATGATGATAATCTACAAAAAGATTTTAAAAGTGCAAAAGATACGATAAAGAAACAAATATCTGAATATATAAAGGACTCAGAGAAATTTAAGAATTTGTTTAATCAAAACCTTATCGATGCTAAAAAAGGGCAAGAGTCAGATTTAATTCTATGGCTAAAGCAATCTAAGGATAATGGTATAGAACTATTTAAAGCCAATAGTGATATCACAGATATAGATGAGGCGTTAGAAATAATCAAATCTTTTAAAGGTTGGACAACTTATTTTAAGGGTTTTCATGAAAATAGAAAAAATGTTTATAGTAGCAATGATATTCCTACATCTATTATTTATAGGATAGTAGATGATAATTTGCCTAAATTTCTAGAAAATAAAGCTAAGTATGAGAGTTTAAAAGACAAAGCTCCAGAAGCTATAAACTATGAACAAATTAAAAAAGATTTGGCAGAAGAGCTAACCTTTGATATTGACTACAAAACATCTGAAGTTAATCAAAGAGTTTTTTCACTTGATGAAGTTTTTGAGATAGCAAACTTTAATAATTATCTAAATCAAAGTGGTATTACTAAATTTAATACTATTATTGGTGGTAAATTTGTAAATGGTGAAAATACAAAGAGAAAAGGTATAAATGAATATATAAATCTATACTCACAGCAAATAAATGATAAAACACTCAAAAAATATAAAATGAGTGTTTTATTTAAGCAAATTTTAAGTGATACAGAATCTAAATCTTTTGTAATTGATAAGTTAGAAGATGATAGTGATGTAGTTACAACGATGCAAAGTTTTTATGAGCAAATAGCAGCTTTTAAAACAGTAGAAGAAAAATCTATTAAAGAAACACTATCTTTATTATTTGATGATTTAAAAGCTCAAAAACTTGATTTGAGTAAAATTTATTTTAAAAATGATAAATCTCTTACTGATCTATCACAACAAGTTTTTGATGATTATAGTGTTATTGGTACAGCGGTACTAGAATATATAACTCAACAAATAGCACCTAAAAATCTTGATAACCCTAGTAAGAAAGAGCAAGAATTAATAGCCAAAAAAACTGAAAAAGCAAAATACTTATCTCTAGAAACTATAAAGCTTGCCTTAGAAGAATTTAATAAGCATAGAGATATAGATAAACAGTGTAGGTTTGAAGAAATACTTGCAAACTTTGCGGCTATTCCGATGATATTTGATGAAATAGCTCAAAACAAAGACAATTTGGCACAGATATCTATCAAATATCAAAATCAAGGTAAAAAAGACCTACTTCAAGCTAGTGCGGAAGATGATGTTAAAGCTATCAAGGATCTTTTAGATCAAACTAATAATCTCTTACATAAACTAAAAATATTTCATATTAGTCAGTCAGAAGATAAGGCAAATATTTTAGACAAGGATGAGCATTTTTATCTAGTATTTGTGGAGTGCTACCTTGAGCTAGCGAATATAGTGCCTCTTTATAACAAAATTAGAAACTATATAACTCAAAAGCCATATAGTGATGAGAAATTTAAGCTCAATTTTGAGAACTCGACTTTGGCTAATGGTTGGGATAAAAATAATGAGCCTGACAATACGGCAATTTTATTTATCAAAGATGATAAATATTATCTGGGTGTGATGAATAAGAAAAATAACAAAATATTTGATGATAAAGCTATCAAAGAAAATAAAGGCGAGGGTTATAAAAAAATTGTTTATAAACTTATACCTGGCGCAAATAAAATGTTACCTCGTGTTTTCTTTTCTGCTAAATCTATAAAATTTTATAATCCTAGTGAAGATATACTTATAATAAGAAATCATTCCACACATACAAAAAATGGTAGTCCTCAAAAAGGATATGAAAAATTTGAGTTTAATATTGAAGATTGCCGAAAATTTATAGATTTTTGTAAACAGTCTATAAGTAAGCATCCGGAGTGGAAAGATTTTGGATTTAGATTTTCTGATACTCAAAGATATAATTCTATAGGTGAATTTTATAGAGAAGTTGAAAATCAAGGCTACAAACTAACTTTTGAAAATATATCAGAGAGCTATATTGATAGCGTAGTTAATCAGGGTAAATTGTACCTATTCCAAATCTATAATAAAGATTTTTCAGCTTATAGCAAAGGGCGACCAAATCTACATACTTTATATTGGAAAGCGCTGTTTGATGAGAGAAATCTTCAAGATGTGGTTTATAAGCTAAATGGTGAGGCAGAGCTTTTTTATCGTAAACAATCAATACCTAAAAAAATCACTCACCCAGCTAAAGAGGCAATAGCTAATAAAAACAAAGATAATCCTAAAAAAGAGAGTGTTTTTGAATATGATTTAATCAAAGATAAACGCTTTACTGAAGATAAGTTTTTCTTTCACTGTCCTATTACAATCAATTTTAAATCTAGTGGAGCTAATAAGTTTAATGATGAAATCAATTTATTGCTAAAAGAAAAAGCAAATGATGTTCATATATTAAGTATAGCAAGAGGTGAAAGACATTTAGCTTACTATACTTTGGTAGATGGTAAAGGCAATATCATCAAACAAGATACTTTCAACATCATTGGTAATGATAGAATGAAAACAAACTACCATGATAAGCTTGCTGCAATAGAGAAAGATAGGGATTCAGCTAGGAAAGACTGGAAAAAGATAAATAACATCAAAGAGATGAAAGAGGGCTATCTATCTCAGGTAGTTCATGAAATAGCTAAGCTAGTTATAGAGTATAATGCTATTGTGGTTTTTGAGGATTTAAATTTTGGATTTAAAAGAGGGCGTTTCAAGGTAGAGAAGCAGGTCTATCAAAAGTTAGAAAAAATGCTAATTGAGAAACTAAACTATCTAGTTTTCAAAGATAATGAGTTTGATAAAACTGGGGGAGTGCTTAGAGCTTATCAGCTAACAGCACCTTTTGAGACTTTTAAAAAGATGGGTAAACAAACAGGTATTATCTACTATGTACCAGCTGGTTTTACTTCAAAAATTTGTCCTGTAACTGGTTTTGTAAATCAGTTATATCCTAAGTATGAAAGTGTCAGCAAATCTCAAGAGTTCTTTAGTAAGTTTGACAAGATTTGTTATAACCTTGATAAGGGCTATTTTGAGTTTAGTTTTGATTATAAAAACTTTGGTGACAAGGCTGCCAAAGGCAAGTGGACTATAGCTAGCTTTGGGAGTAGATTGATTAACTTTAGAAATTCAGATAAAAATCATAATTGGGATACTCGAGAAGTTTATCCAACTAAAGAGTTGGAGAAATTGCTAAAAGATTATTCTATCGAATATGGGCATGGCGAATGTATCAAAGCAGCTATTTGCGGTGAGAGCGACAAAAAGTTTTTTGCTAAGCTAACTAGTGTCCTAAATACTATCTTACAAATGCGTAACTCAAAAACAGGTACTGAGTTAGATTATCTAATTTCACCAGTAGCAGATGTAAATGGCAATTTCTTTGATTCGCGACAGGCGCCAAAAAATATGCCTCAAGATGCTGATGCCAATGGTGCTTATCATATTGGGCTAAAAGGTCTGATGCTACTAGGTAGGATCAAAAATAATCAAGAGGGCAAAAAACTCAATTTGGTTATCAAAAATGAAGAGTATTTTGAGTTCGTGCAGAATAGGAATAACTCTGGTGGTTCTTCTGGTGGTTCTACTAATCTGTCAGATATTATTGAAAAGGAGACCGGTAAGCAACTGGTTATCCAGGAATCCATCCTCATGCTCCCAGAGGAGGTGGAAGAAGTCATTGGGAACAAGCCGGAAAGCGATATACTCGTGCACACCGCCTACGACGAGAGCACCGACGAGAATGTCATGCTTCTGACTAGCGACGCCCCTGAATACAAGCCTTGGGCTCTGGTCATACAGGATAGCAACGGTGAGAACAAGATTAAGATGCTCTAG

**6. pTAC-Apobec1-denAsCpf1-UGI**

Apobec1 in bold blue, denAsCpf1 in black, UGI in orange, linker sequences in gray

ATGAGCTCAGAGACTGGCCCAGTGGCTGTGGACCCCACATTGAGACGGCGGATCGAGCCCCATGAGTTTGAGGTATTCTTCGATCCGAGAGAGCTCCGCAAGGAGACCTGCCTGCTTTACGAAATTAATTGGGGGGGCCGGCACTCCATTTGGCGACATACATCACAGAACACTAACAAGCACGTCGAAGTCAACTTCATCGAGAAGTTCACGACAGAAAGATATTTCTGTCCGAACACAAGGTGCAGCATTACCTGGTTTCTCAGCTGGAGCCCATGCGGCGAATGTAGTAGGGCCATCACTGAATTCCTGTCAAGGTATCCCCACGTCACTCTGTTTATTTACATCGCAAGGCTGTACCACCACGCTGACCCCCGCAATCGACAAGGCCTGCGGGATTTGATCTCTTCAGGTGTGACTATCCAAATTATGACTGAGCAGGAGTCAGGATACTGCTGGAGAAACTTTGTGAATTATAGCCCGAGTAATGAAGCCCACTGGCCTAGGTATCCCCATCTGTGGGTACGACTGTACGTTCTTGAACTGTACTGCATCATACTGGGCCTGCCTCCTTGTCTCAACATTCTGAGAAGGAAGCAGCCACAGCTGACATTCTTTACCATCGCTCTTCAGTCTTGTCATTACCAGCGACTGCCCCCACACATTCTCTGGGCCACCGGGTTGAAAAGCGGCAGCGAGACTCCCGGGACCTCAGAGTCCGCCACACCCGAAAGTATGACACAGTTCGAGGGCTTTACCAATCTGTACCAGGTGTCCAAGACCCTGAGGTTCGAGCTGATCCCTCAGGGCAAGACCCTGAAGCACATCCAGGAGCAGGGCTTTATCGAGGAGGACAAGGCCAGAAATGACCACTACAAGGAGCTGAAGCCCATCATCGACAGAATCTACAAGACATACGCCGACCAGTGTCTGCAGCTGGTGCAGCTGGACTGGGAGAACCTGAGCGCCGCCATCGACTCCTACAGAAAGGAGAAGACAGAGGAGACAAGGAATGCCCTGATCGAGGAGCAGGCCACCTACAGGAACGCCATCCACGACTACTTCATCGGCAGAACAGACAATCTGACAGACGCCATCAATAAGAGACACGCCGAGATCTACAAGGGCCTGTTCAAGGCCGAGCTGTTCAATGGCAAGGTGCTGAAGCAGCTGGGCACCGTGACCACAACCGAGCACGAGAATGCCCTGCTGAGGAGCTTTGATAAGTTCACCACATACTTTTCCGGCTTTTACAGGAATAGAAAGAATGTGTTTAGCGCCGAGGATATCAGCACCGCCATCCCTCACAGAATCGTGCAGGACAATTTCCCCAAGTTTAAGGAGAACTGTCACATCTTCACCAGACTGATCACAGCCGTGCCCTCCCTGAGGGAGCACTTCGAGAACGTGAAGAAGGCCATCGGCATCTTCGTGAGCACCTCCATCGAGGAGGTGTTCAGCTTCCCTTTTTACAACCAGCTGCTGACCCAGACACAGATCGACCTGTACAACCAGCTCCTGGGCGGCATCAGCAGAGAGGCCGGCACAGAGAAGATCAAGGGCCTGAACGAGGTGCTGAACCTGGCCATCCAGAAGAATGACGAGACAGCCCACATCATCGCCTCCCTGCCTCACAGGTTCATCCCCCTGTTCAAGCAGATCCTGAGCGATAGGAATACCCTGAGCTTTATCCTGGAGGAGTTTAAGAGCGACGAGGAGGTGATCCAGTCCTTTTGTAAGTACAAGACACTGCTGAGAAATGAGAACGTGCTGGAGACAGCCGAGGCCCTGTTCAATGAGCTGAACTCCATCGATCTGACCCACATCTTCATCAGCCACAAGAAGCTGGAGACAATCAGCAGCGCCCTGTGCGACCACTGGGATACCCTGAGGAACGCCCTGTACGAGAGGAGGATCTCCGAGCTGACAGGCAAGATCACAAAGTCCGCCAAGGAGAAGGTGCAGAGAAGCCTGAAGCACGAGGACATCAATCTGCAGGAGATCATCTCCGCCGCCGGCAAGGAGCTGTCCGAGGCTTTCAAGCAGAAGACAAGCGAGATCCTGTCCCACGCCCACGCCGCCCTGGACCAACCTCTGCCTACAACCCTGAAGAAGCAGGAGGAGAAGGAGATCCTGAAGTCCCAGCTGGACTCCCTGCTGGGCCTGTACCACCTGCTGGATTGGTTCGCCGTGGACGAGTCCAACGAGGTGGACCCCGAGTTTTCCGCCAGGCTGACAGGCATCAAGCTGGAGATGGAGCCCTCCCTGTCCTTTTACAACAAGGCCAGAAACTACGCCACCAAGAAGCCTTACTCCGTGGAGAAGTTCAAGCTGAACTTTCAGATGCCCACACTGGCCAGAGGCTGGGATGTGAATAGAGAGAAGAATAATGGCGCCATCCTGTTCGTGAAGAACGGCCTGTACTACCTGGGCATCATGCCCAAGCAGAAGGGCAGATACAAGGCCCTGTCCTTCGAGCCCACCGAGAAGACCTCCGAGGGCTTCGACAAGATGTACTACGATTACTTCCCTGACGCCGCCAAGATGATCCCTAAGTGCTCCACCCAGCTGAAGGCCGTGACAGCCCACTTCCAGACACACACAACCCCCATCCTGCTGTCCAATAATTTCATCGAGCCTCTGGAGATCACAAAGGAGATCTACGATCTGAACAATCCCGAGAAGGAGCCCAAGAAGTTCCAGACCGCCTACGCCAAGAAGACAGGCGACCAGAAGGGCTACAGGGAGGCCCTGTGTAAGTGGATCGACTTCACCAGAGACTTTCTGTCCAAGTACACAAAGACCACCAGCATCGACCTGTCCTCCCTGAGGCCTAGCTCCCAGTACAAGGACCTGGGCGAGTACTACGCCGAGCTGAACCCTCTGCTGTACCACATCAGCTTTCAGAGAATCGCCGAGAAGGAGATCATGGATGCCGTGGAGACAGGCAAGCTGTACCTGTTCCAGATCTACAATAAGGACTTCGCCAAGGGCCACCACGGCAAGCCTAACCTGCACACCCTGTACTGGACCGGCCTGTTTTCCCCTGAGAACCTGGCCAAGACCTCCATCAAGCTGAATGGCCAGGCCGAGCTGTTTTACAGGCCTAAGTCCAGAATGAAGAGAATGGCCCACAGACTGGGCGAGAAGATGCTGAATAAGAAGCTGAAGGACCAGAAGACCCCTATCCCTGACACCCTGTACCAGGAGCTGTACGACTACGTGAATCACAGACTGTCCCACGATCTGTCCGATGAGGCCAGGGCCCTGCTGCCCAATGTGATCACCAAGGAGGTGTCCCACGAGATCATCAAGGACAGAAGGTTTACCAGCGACAAGTTCTTTTTTCACGTGCCCATCACCCTGAACTACCAGGCCGCCAATTCCCCCAGCAAGTTCAATCAGAGAGTGAATGCCTACCTGAAGGAGCACCCTGAGACACCTATCATCGGCATCGCCAGGGGCGAGAGGAACCTGATCTACATCACAGTGATCGACAGCACCGGCAAGATCCTGGAGCAGAGAAGCCTGAATACAATCCAGCAGTTTGATTACCAGAAGAAGCTGGACAATAGGGAGAAGGAGAGGGTGGCCGCCAGACAGGCCTGGAGCGTGGTGGGAACCATCAAGGACCTGAAGCAGGGCTACCTGAGCCAGGTGATCCACGAGATCGTGGACCTGATGATCCACTACCAGGCCGTGGTGGTGCTGGAGAATCTGAACTTTGGCTTCAAGAGCAAGAGAACAGGCATCGCCGAGAAAGCCGTGTACCAGCAGTTTGAGAAGATGCTCATCGACAAGCTGAACTGTCTGGTGCTGAAGGACTACCCTGCCGAGAAGGTGGGCGGCGTGCTGAACCCTTACCAGCTGACCGATCAGTTTACATCCTTTGCCAAGATGGGCACCCAGAGCGGCTTTCTGTTCTACGTGCCCGCCCCCTACACCTCCAAGATCGACCCCCTGACCGGCTTCGTGGATCCCTTTGTGTGGAAGACCATCAAGAACCACGAGTCCAGGAAGCACTTCCTGGAGGGCTTCGATTTTCTGCACTACGATGTGAAGACCGGCGATTTTATCCTGCACTTTAAGATGAATAGGAATCTGAGCTTTCAGAGGGGCCTGCCCGGCTTCATGCCTGCCTGGGACATCGTGTTTGAGAAGAATGAGACACAGTTTGATGCCAAGGGCACCCCTTTCATCGCCGGCAAGAGAATCGTGCCCGTGATCGAGAACCACAGATTCACAGGCAGGTACAGAGACTTGTACCCCGCCAACGAGCTGATCGCCCTGCTGGAGGAGAAGGGCATCGTGTTCAGGGATGGCAGCAATATCCTGCCTAAGCTGCTGGAGAACGACGACAGCCACGCCATCGATACAATGGTGGCCCTGATCAGATCCGTGCTGCAGATGAGGAACAGCAACGCCGCCACCGGCGAGGATTACATCAACAGCCCTGTGAGGGATCTGAACGGCGTGTGCTTCGACAGCAGATTCCAGAACCCTGAGTGGCCTATGGATGCCGACGCCAATGGCGCCTACCACATCGCCCTGAAGGGCCAGCTGCTGCTGAATCACCTGAAGGAGTCCAAGGACCTGAAACTGCAGAACGGCATCAGCAACCAGGATTGGCTGGCCTACATCCAGGAGCTGAGGAACCTCTGGTGGTTCTTCTGGTGGTTCTACTAATCTGTCAGATATTATTGAAAAGGAGACCGGTAAGCAACTGGTTATCCAGGAATCCATCCTCATGCTCCCAGAGGAGGTGGAAGAAGTCATTGGGAACAAGCCGGAAAGCGATATACTCGTGCACACCGCCTACGACGAGAGCACCGACGAGAATGTCATGCTTCTGACTAGCGACGCCCCTGAATACAAGCCTTGGGCTCTGGTCATACAGGATAGCAACGGTGAGAACAAGATTAAGATGCTCTAG

**Supplement Table 2. Oligonucleotides used in this study**

**For plasmid construction**

| Sequences Description | 5’ 🡪 3’ |
| --- | --- |
| Forward PCR primer to amplify *dfncpf1* gene | aaagaggagaaaatgtcaatttatcaagaatttgtta |
| Reverse PCR primer to amplify *dfncpf1* gene | gatgcctggactagtactagttattcctattctgcacgaactcaaa |
| Forward PCR primer to amplify RGP backbone | tactagtccaggcatcaaataaaacgaaagg |
| Reverse PCR primer to amplify RGP backbone | cattttctcctctttttaaacaaaattatttgtagaggc |
| Forward PCR primer to construct PAM library | tgtcaacggtcataannnccgtgcgtggcgagggtgaaggcgcaactaat |
| Reverse PCR primer to construct PAM library | ttatgaccgttgacatcaccatccagt |
| Forward PCR primer to construct crRNA plasmid | gtggcgagggtgaaggtgagtctaagaactttaaataatttctactgttgtagatgaagcttgggcccgaacaaa |
| Reverse PCR primer to construct crRNA plasmid | ccttcaccctcgccacgcacgatctacaacagtagaaattatttaaagttcttagacactagtattatacctaggac |
| Forward PCR primer for error-prone PCR | tcaaaacaaagacaatttggcacagatatctatcaaatatcaaaat |
| Reverse PCR primer for error-prone PCR | ccctgattaactacgctatcaatatagct |
| Forward PCR primer to amplify *apobec1* gene | ttgtttaaaaagaggagaaaatgagctcagagactggcccag |
| Reverse PCR primer to amplify *apobec1* gene | gcggactctgaggtcccgggagtctcgctgccgcttttcaacccggtggcccaga |
| Forward PCR primer to amplify *gui* gene | aactctggtggttcttctggtggttctactaatctgtcagatatt |
| Reverse PCR primer to amplify *gui* gene | gatgcctggactagtactagagcatcttaatcttgttctcaccg |
| Forward PCR primer to amplify *denascpf1* gene | gggacctcagagtccgccacacccgaaagtatgacacagttcgagggctttacc |
| Reverse PCR primer to amplify *denascpf1* gene | ccagaagaaccaccagagttcctcagctcctggatgtagg |

**Key oligos used in this study**

| Primers Description | 5’ 🡪 3’ |
| --- | --- |
| Target site for YFP repression in negative screen | cgtgcgtggcgagggtgaagg |
| Target site for YFP shutdown via base editing | gtgatcgacagcaacaagtga |
| Target site for base editing with different PAMs | gggcactctccagatagggat |
| Full repeat of FnCpf1 crRNA used in this study | gtctaagaactttaaataatttctactgttgtagat |
| Full repeat of enAsCpf1 crRNA used in this study | gtcaaaagacctttttaatttctactcttgtagat |
| *galK*-T1 targeted for base editing in *E.coli* genome | gcgctgatcccggaagagctg |
| *galK*-T2 targeted for base editing in *E.coli* genome | cgcgctcggcaagaaagatca |
| *galK*-T3 targeted for base editing in *E.coli* genome | aaattgacactctggtagaaa |
| *gsiA*-T4 targeted for base editing in *E.coli* genome | agcgaatcgataccttgtcac |
| *gsiA*-T5 targeted for base editing in *E.coli* genome | gttatctttatcactcacgat |
| *ycbF*-T6 targeted for base editing in *E.coli* genome | accgtgtcatgtcgcctggcg |

**For deep sequencing used in base editing experiments**

| Primers Description | 5’ 🡪 3’ |
| --- | --- |
| Forward PCR primer for deep sequencing of base editing in shutdown YFP in Figure 3C | gtgacgcaactaatggttaa |
| Reverse PCR primer for deep sequencing of base editing in shutdown YFP in Figure 3C | atgggcaccaccccggtgaa |
| Forward PCR primer for deep sequencing of base editing with different PAMs in Figure 3D | gcccttagtgactcgaattcgaattc |
| Reverse PCR primer for deep sequencing of base editing with different PAMs in Figure 3D | gctcctgcgaaagtcgggt |
| Forward PCR primer for deep sequencing of target site *galK*-T1 in Figure 4 | tacgcatgaccggcggcgga |
| Reverse PCR primer for deep sequencing of target site *galK*-T1 in Figure 4 | tttgcttcatattgttcagcgacag |
| Forward PCR primer for deep sequencing of target site *galK*-T2 in Figure 4 | tgtaggctgtaactgcggga |
| Reverse PCR primer for deep sequencing of target site *galK*-T2 in Figure 4 | gctttggtccccagtgagcg |
| Forward PCR primer for deep sequencing of target site *galK*-T3 in Figure 4 | atgcctctatgcgcgatgat |
| Reverse PCR primer for deep sequencing of target site *galK*-T3 in Figure 4 | tgcgtacgccacctttgtcg |
| Forward PCR primer for deep sequencing of target site *gsiA*-T4 in Figure 4 | tggtcgaatcgcagggcggc |
| Reverse PCR primer for deep sequencing of target site *gsiA*-T4 in Figure 4 | tgaaaaataaactgaatatcccggcgta |
| Forward PCR primer for deep sequencing of target site *gsiA*-T5 in Figure 4 | agatcctgcaattaatcaaagtattgca |
| Reverse PCR primer for deep sequencing of target site *gsiA*-T5 in Figure 4 | accagtacccgatcggcaat |
| Forward PCR primer for deep sequencing of target site *ycbF*-T6 in Figure 4 | cctaaaagccagcgtcagacaa |
| Reverse PCR primer for deep sequencing of target site *ycbF*-T6 in Figure 4 | ccttctttgcactcaacccg |

**Supplement Table 3. All dFnCpf1 variants screened from the four rounds of directed evolution**

| **Mutants evolved from the PAM CCCC pathway** | **Repression fold** |
| --- | --- |
| E566V/K671R/D751G (refer to VRG) | 51.22 |
| VRG/E635K | 65.20 |
| VRG/N580H | 63.17 |
| VRG /N508H/F570L | 81.92 |
| VRG/N508H/F570L/E752V | 91.30 |
| VRG/N508H/F570L/N637S | 116.43 |
| VRG/N508H/F570L/N553D | 102.18 |
| VRG/N508H/F570L/I542V | 93.26 |
| VRG/N508H/F570L/I542V/E559G | 102.77 |
| **Mutants evolved from the PAM GCCC pathway** | |
| VRG | 20.34 |
| VRG/F570L | 37.60 |
| VRG/E635K | 37.53 |
| VRG/F570L/E756G | 36.08 |
| VRG/F570L/N634D | 40.90 |
| VRG/F570L/ D687N | 57.09 |
| VRG/F570L/N634D/K611R | 61.30 |
| VRG/F570L/N634D/R755K | 61.84 |
| VRG/F570L/N634D/E767G | 54.26 |
| **Mutants evolved from the PAM CCGC pathway** | |
| VRG/S518G | 155.27 |
| VRG/S546R | 143.85 |
| VRG/L537I | 33.01 |
| VRG/S518G/K639R | 159.86 |
| VRG/S518G/K639R/K719R | 135.95 |
| VRG/L537I/K639R/F565L | 140.00 |
| VRG/L537I/F565L/K639R/K719R | 143.32 |
| VRG/L537I/F565L/N634D/K719R | 151.20 |
| **Mutants evolved from the PAM GCGC pathway** | |
| VRG/F570L | 68.77 |
| VRG/S546R | 62.69 |
| VRG/F570L/E686D | 77.17 |
| VRG/F570L/N634D/E686D | 68.68 |
| VRG/F570L/I575M/E686D | 63.22 |
| VRG/F570L/N634D/L572I | 63.23 |
| VRG/F570L/N634D/F596Y | 68.05 |
| **Mutants evolved from the PAM CGCC pathway** | |
| VRG/K613N (refer to VRGN) | 10.87 |
| VRGN/F570L | 27.31 |
| VRGN/F570L/N637S | 23.25 |
| VRGN/N534K/N637S | 20.39 |
| VRGN/N637S/N534K/G664V | 64.75 |
| VRGN/N637S/N534K/G664S | 38.40 |
| **Mutants evolved from the PAM GGCC pathway** | |
| VRGN/F570L/G664S | 35.06 |
| VRGN/F570L/G664S/N637Y | 62.14 |
| VRGN/F570L/G664S/F710S | 29.30 |
| VRGN/F570L/G664S/K647R/I728L | 40.78 |
| VRGN/F570L/G664S/A619V/S729R | 36.03 |
| **Mutants evolved from the PAM CGGC pathway** | |
| VRGN/Y724C | 32.83 |
| VRGN/Y724C/F570L | 47.10 |
| VRGN/Y724C/D522V/K650R | 33.19 |
| VRGN/Y724C/D616N | 33.22 |
| VRGN/Y724C/K505N/F570L | 42.95 |
| VRGN/Y724C/F570L/I582V | 42.26 |
| VRGN/Y724C/F570L/E715D | 38.75 |
| **Mutants evolved from the PAM GGGC pathway** | |
| VRGN/Y724C | 15.37 |
| VRGN/Y724C/Q509R | 32.09 |
| VRGN/Y724C/F570L | 36.37 |
| VRGN/Y724C/F570L/R690I/L662I | 45.41 |
| VRGN/Y724C/F570L/R690I /D625E | 41.59 |

**Supplement Table 4. PAM preference analysis profile data. YFP fluorescence intensity after 200 µM IPTG induction was used as the characterization value.**

| **PAM** | WT | CCCC-M1  (VRG/N508H  /N637S) | GCCC-M1  (VRG/F570L/  N634D/R755K) | CCGC-M1  (VRG/S518G  /K639R) | GCGC-M1  (VRG/F570L  /E686D) |
| --- | --- | --- | --- | --- | --- |
| **AAAC** | 3839.2 | 3921.2 | 3606.6 | 4136 | 3960.8 |
| **ATAC** | 2832.4 | 989.4 | 670.8 | 1287.2 | 1092.6 |
| **ACAC** | 2891.4 | 251.4 | 199.8 | 425.8 | 318.2 |
| **AGAC** | 4170.4 | 4149.2 | 3872.4 | 4457.8 | 4328.6 |
| **TAAC** | 3818.4 | 4167.4 | 3193.8 | 4063.2 | 3946.6 |
| **TTAC** | 1284.6 | 217.6 | 140.4 | 274 | 203.4 |
| **TCAC** | 3186.8 | 404.8 | 252.6 | 489.2 | 378.2 |
| **TGAC** | 3927 | 4490.4 | 4171.6 | 4264.4 | 4294.8 |
| **CAAC** | 4158 | 4275.6 | 3535.6 | 4252.4 | 4145.6 |
| **CTAC** | 1851.8 | 262.8 | 172.4 | 393 | 292 |
| **CCAC** | 3599 | 306.2 | 182.8 | 387.8 | 291.4 |
| **CGAC** | 4281.2 | 4429.4 | 3865.2 | 4561.4 | 4219.4 |
| **GAAC** | 4430.4 | 4945.2 | 4512 | 4609 | 4669.6 |
| **GTAC** | 2943.8 | 817 | 471.8 | 1433 | 1600.2 |
| **GCAC** | 2010.8 | 146 | 93.06 | 209.4 | 153.6 |
| **GGAC** | 4396 | 4816.8 | 4538.8 | 4545.4 | 4584.4 |
| **AATC** | 53.32 | 9.72 | 7.1 | 18 | 8.64 |
| **ATTC** | 31.78 | 33.92 | 30.02 | 41.46 | 34.42 |
| **ACTC** | 115.8 | 31.22 | 21.84 | 32.56 | 26.5 |
| **AGTC** | 2956.2 | 2154 | 1173.2 | 2302.4 | 2418 |
| **TATC** | 2113.2 | 965.4 | 516.8 | 1258 | 1004 |
| **TTTC** | 9.56 | 19.04 | 14.56 | 36.7 | 16.46 |
| **TCTC** | 39.56 | 22.94 | 16.22 | 18.74 | 16.82 |
| **TGTC** | 2064.4 | 1902.2 | 1507.4 | 2330.6 | 2227 |
| **CATC** | 3298.4 | 1272.4 | 694 | 1643.6 | 1534.8 |
| **CTTC** | 24.04 | 41.24 | 33.38 | 55.62 | 37.08 |
| **CCTC** | 130 | 32.96 | 29.92 | 42.06 | 32.62 |
| **CGTC** | 3404.2 | 2714.6 | 2040 | 3260 | 3029.6 |
| **GATC** | 2673.6 | 982.2 | 612.6 | 1516.4 | 1387.8 |
| **GTTC** | 16.3 | 33.16 | 23.68 | 42.02 | 36.44 |
| **GCTC** | 74.1 | 25.28 | 24.04 | 30.72 | 26.34 |
| **GGTC** | 2458.8 | 3046.6 | 2279.2 | 3140.4 | 3041.2 |
| **AACC** | 3759.6 | 755.4 | 350.6 | 988.8 | 758.2 |
| **ATCC** | 1551.6 | 322.2 | 155 | 379 | 258 |
| **ACCC** | 912.6 | 43.26 | 40.5 | 71.76 | 50.56 |
| **AGCC** | 3878.4 | 3129.4 | 2253.6 | 3450.8 | 3125.6 |
| **TACC** | 3527.6 | 1287.6 | 733.4 | 1640.6 | 1607.4 |
| **TTCC** | 50.1 | 32.42 | 26.3 | 49.24 | 33.06 |
| **TCCC** | 186.4 | 16.62 | 14.8 | 21.5 | 16.94 |
| **TGCC** | 4192.6 | 4230.4 | 3545.6 | 3401.6 | 4312 |
| **CACC** | 3403.4 | 537.8 | 236.6 | 787.6 | 516.2 |
| **CTCC** | 303.6 | 76.36 | 57.4 | 113.4 | 85.08 |
| **CCCC** | 816 | 37.14 | 30.4 | 51.68 | 37.68 |
| **CGCC** | 4079.2 | 3433.8 | 2528.6 | 3728.6 | 3042.6 |
| **GACC** | 3428.6 | 876.4 | 447.6 | 1187.8 | 888.6 |
| **GTCC** | 224 | 108.72 | 66.48 | 137 | 116.2 |
| **GCCC** | 466.2 | 31.2 | 27.66 | 99.44 | 34 |
| **GGCC** | 3518 | 3423 | 2746.2 | 3416.6 | 3416.2 |
| **AAGC** | 3882.4 | 3735 | 2621.6 | 3779.8 | 3554.4 |
| **ATGC** | 3581.8 | 1727.8 | 1222.2 | 2032.2 | 1813.8 |
| **ACGC** | 2907.8 | 135 | 84.62 | 208.8 | 141.2 |
| **AGGC** | 3815 | 4128.8 | 3860.2 | 4174.6 | 3920 |
| **TAGC** | 4256.2 | 2930.6 | 2195 | 3358.6 | 3257.2 |
| **TTGC** | 2314.8 | 392.6 | 360 | 626.4 | 493.8 |
| **TCGC** | 1421.6 | 46.04 | 179.46 | 75.3 | 59.62 |
| **TGGC** | 3952.6 | 4305.8 | 4220.2 | 4268.4 | 4209.8 |
| **CAGC** | 3824.8 | 3586.8 | 2651.2 | 3710.2 | 3661 |
| **CTGC** | 2276 | 260 | 154 | 376 | 326 |
| **CCGC** | 2731.6 | 220.8 | 164.6 | 37.74 | 69.88 |
| **CGGC** | 4255 | 4384.4 | 3914.2 | 4349.4 | 4288.8 |
| **GAGC** | 4204.2 | 3954.8 | 3281.2 | 4179.4 | 4135.6 |
| **GTGC** | 1814.2 | 268 | 228 | 451.4 | 356.4 |
| **GCGC** | 1769.8 | 44.76 | 53.5 | 96.02 | 68.16 |
| **GGGC** | 3845.5 | 4366.6 | 4116.6 | 4299.4 | 4276.6 |

| **PAM** | CGCC-M1  (VRGN/N637S  /N534K/G664V) | GGCC-M1  （VRGN/F570L  /G664S/N637Y） | CGGC-M1  (VRGN/Y724C  /F570L) | GGGC-M1  (VRGN/Y724C/  F570L/R690I/L662I) |
| --- | --- | --- | --- | --- |
| **AAAC** | 2510.88 | 3513.4 | 2033 | 2045 |
| **ATAC** | 164.80 | 508.2 | 233.4 | 176.8 |
| **ACAC** | 327.60 | 1224 | 386.6 | 359 |
| **AGAC** | 212.20 | 594.2 | 302.4 | 196 |
| **TAAC** | 246.60 | 767 | 506 | 390.2 |
| **TTAC** | 32.10 | 57.5 | 33.12 | 30.26 |
| **TCAC** | 1343.20 | 2327.4 | 345.2 | 337 |
| **TGAC** | 136.80 | 361.6 | 139.8 | 123.4 |
| **CAAC** | 1113.33 | 2816.4 | 1160.6 | 1296.8 |
| **CTAC** | 34.76 | 57.5 | 35.46 | 27.32 |
| **CCAC** | 863.00 | 1611.6 | 200 | 159 |
| **CGAC** | 85.14 | 136.8 | 64.56 | 63.58 |
| **GAAC** | 3166.40 | 4037.4 | 2040.6 | 1605.2 |
| **GTAC** | 710.60 | 673.8 | 114 | 125.8 |
| **GCAC** | 118.20 | 260.2 | 103 | 93.48 |
| **GGAC** | 806.60 | 1350.6 | 296.2 | 239 |
| **AATC** | 9.70 | 11.62 | 10.1 | 5.62 |
| **ATTC** | 39.18 | 36.62 | 38.26 | 26.22 |
| **ACTC** | 67.82 | 81.28 | 61.6 | 49.16 |
| **AGTC** | 82.08 | 88.56 | 74.68 | 51.16 |
| **TATC** | 32.70 | 36.06 | 47.28 | 32.92 |
| **TTTC** | 19.26 | 16.14 | 17.26 | 14.38 |
| **TCTC** | 35.06 | 28.38 | 28 | 20.92 |
| **TGTC** | 66.53 | 71.04 | 72.66 | 46.4 |
| **CATC** | 47.68 | 48.96 | 60.8 | 34.94 |
| **CTTC** | 54.80 | 48.14 | 27.36 | 30.36 |
| **CCTC** | 82.72 | 125.2 | 65.92 | 66.42 |
| **CGTC** | 64.56 | 71.82 | 63.86 | 42.58 |
| **GATC** | 75.42 | 143.6 | 96.14 | 93.44 |
| **GTTC** | 33.46 | 29.86 | 36.6 | 23.94 |
| **GCTC** | 38.00 | 39.98 | 41.66 | 30.62 |
| **GGTC** | 71.16 | 81.08 | 84.8 | 53.56 |
| **AACC** | 88.08 | 154 | 135.4 | 75.42 |
| **ATCC** | 109.92 | 148.2 | 173 | 88.66 |
| **ACCC** | 150.60 | 144 | 164.8 | 102.4 |
| **AGCC** | 117.00 | 153.4 | 168.4 | 104.6 |
| **TACC** | 157.40 | 320 | 207.8 | 254.6 |
| **TTCC** | 36.28 | 31.2 | 34.14 | 26.32 |
| **TCCC** | 34.16 | 32.12 | 35.42 | 30.56 |
| **TGCC** | 116.14 | 127.4 | 119.4 | 113 |
| **CACC** | 150.80 | 237 | 65.12 | 58.54 |
| **CTCC** | 37.16 | 36.12 | 44.12 | 32.72 |
| **CCCC** | 128.00 | 170 | 133.2 | 141.2 |
| **CGCC** | 69.64 | 84.72 | 98.8 | 67.08 |
| **GACC** | 46.74 | 59.4 | 82.2 | 53.28 |
| **GTCC** | 56.98 | 72.36 | 93.12 | 54.84 |
| **GCCC** | 57.66 | 58.96 | 70.4 | 46.44 |
| **GGCC** | 100.63 | 99.8 | 121.2 | 80.42 |
| **AAGC** | 2551.80 | 3412.8 | 993.4 | 673.6 |
| **ATGC** | 1331.00 | 2340.8 | 1357 | 1442.6 |
| **ACGC** | 177.60 | 341 | 210.6 | 152.2 |
| **AGGC** | 2070.00 | 3179.4 | 1979.2 | 1812 |
| **TAGC** | 220.40 | 615.8 | 281.8 | 183.2 |
| **TTGC** | 483.80 | 667.8 | 165.6 | 155 |
| **TCGC** | 68.92 | 109.6 | 73.04 | 44.4 |
| **TGGC** | 140.20 | 265.4 | 148.2 | 101.6 |
| **CAGC** | 1877.40 | 2750.6 | 577.4 | 512 |
| **CTGC** | 41.36 | 48 | 35.6 | 26.4 |
| **CCGC** | 17.85 | 979.2 | 45.12 | 6.38 |
| **CGGC** | 102.94 | 144.2 | 90.14 | 62.66 |
| **GAGC** | 1309.60 | 2915.4 | 1528 | 1560.2 |
| **GTGC** | 79.08 | 84.6 | 64.8 | 45.42 |
| **GCGC** | 81.66 | 127.2 | 75.46 | 54.4 |
| **GGGC** | 141.40 | 204.4 | 146.2 | 121.6 |
